# Mesenchymal stromal cells restrain the Th17 cell response via L-amino-acid oxidase within lymph nodes

**DOI:** 10.1038/s41419-024-07024-7

**Published:** 2024-09-02

**Authors:** Qi Ni, Le Zhen, Zhu Zeng, Jingwen Yang, Yukai Wang, Huanke Xu, Qixiang Zhang, Yongcheng Zhu, Yu Tao, Jing Wang, Qing Liu, Kezheng Yi, Yang Chen, Qian Chen, Guangji Wang, Fang Zhou, Yunlong Shan

**Affiliations:** 1 Key Laboratory of Drug Metabolism and Pharmacokinetics, Haihe Laboratory of Cell Ecosystem, State Key Laboratory of Natural Medicines, China Pharmaceutical University, Nanjing, China; 2 Genetic Skin Disease Center, Jiangsu Key Laboratory of Molecular Biology for Skin Diseases and STIs, Institute of Dermatology, Chinese Academy of Medical Sciences and Peking Union Medical College, Nanjing, China; 3 Jiangsu Province Hospital of Chinese Medicine, The Affiliated Hospital of Nanjing University of Chinese Medicine, Nanjing, China; 4 Jiangsu Renocell Biotech Co. Ltd., Nanjing, China

**Keywords:** Lymph node, Psoriasis, Mesenchymal stem cells

## Abstract

Mesenchymal stromal/stem cells (MSC) have emerged as a promising therapeutic avenue for treating autoimmune diseases, eliciting considerable interest and discussion regarding their underlying mechanisms. This study revealed the distinctive ability of human umbilical cord MSC to aggregate within the lymph nodes of mice afflicted with autoimmune diseases, but this phenomenon was not observed in healthy mice. The specific distribution is driven by the heightened expression of the CCL21-CCR7 axis in mice with autoimmune diseases, facilitating the targeted homing of MSC to the lymph nodes. Within the lymph nodes, MSC exhibit a remarkable capacity to modulate Th17 cell function, exerting a pronounced anti-inflammatory effect. Transplanted MSC stimulates the secretion of L-amino-acid oxidase (LAAO), a response triggered by elevated levels of tumor necrosis factor-α (TNF-α) in mice with autoimmune diseases through the NF-κB pathway. The presence of LAAO is indispensable for the efficacy of MSC, as it significantly contributes to the inhibition of Th17 cells. Furthermore, LAAO-derived indole-3-pyruvic acid (I3P) serves as a potent suppressor of Th17 cells by activating the aryl hydrocarbon receptor (AHR) pathway. These findings advance our understanding of the global immunomodulatory effects exerted by MSC, providing valuable information for optimizing therapeutic outcomes.

## Introduction

Autoimmune disease arises from deficiencies in immunological self-tolerance, leading to chronic inflammation and tissue damage [1]. With over 80 known conditions affecting 5–8% of the population, the incidence and prevalence of autoimmune diseases continue to increase [2]. Despite treatments such as immunosuppressive drugs and antibodies [2, 3], a comprehensive cure remains elusive. Thus, there is an urgent need for therapies capable of restoring immune homeostasis and providing lasting symptom relief.

Mesenchymal stromal/stem cells (MSC) offer potential therapeutic benefits for various immune-related diseases due to their immunosuppressive, self-renewing, and multi-differentiation properties [4]. MSC therapy has proven effective in treating psoriasis [5], systemic lupus erythematosus (SLE) [6], inflammatory bowel disease (IBD) [7], and graft-versus-host disease (GvHD) [8]. Through the expression of regulatory molecules, MSC can directly inhibit the activation and survival of T and B cells [9, 10]. Additionally, MSC suppress the differentiation of inflammatory T cell subsets while promoting regulatory T cell development by the secretion of immunomodulatory factors [11]. Moreover, MSC indirectly modulate the immune response by constraining dendritic cell (DC) maturation and activation, thereby inhibiting T-cell activation [9].

The prerequisite for the immunomodulatory efficacy of MSC is their specific migration and persistence at the injury site [12]. However, existing evidence indicates that MSC are rapidly cleared in normal mice after systemic infusion, and primarily entrapped by the pulmonary system [13, 14]. This mismatch between swift elimination and the anticipated therapeutic benefits for autoimmune disease highlights a critical knowledge gap, emphasizing the need for the study of MSC tissue distribution in mice during pathological states.

In our study, we investigated the distribution pattern of MSC within mice with autoimmune diseases and elucidated the mechanism by which MSC migrated to the lymph nodes. Remarkably, MSC exhibited a superior ability to suppress the Th17 cell response and regulate immune homeostasis, mediated by L-amino-acid oxidase (LAAO). The targeted homing of MSC to lymph nodes and the LAAO secretion may elucidate the therapeutic efficacy in treating autoimmune diseases.

## Results

### MSC exhibit a homing tendency toward lymph nodes in mice with autoimmune diseases

**Fig. 1:**
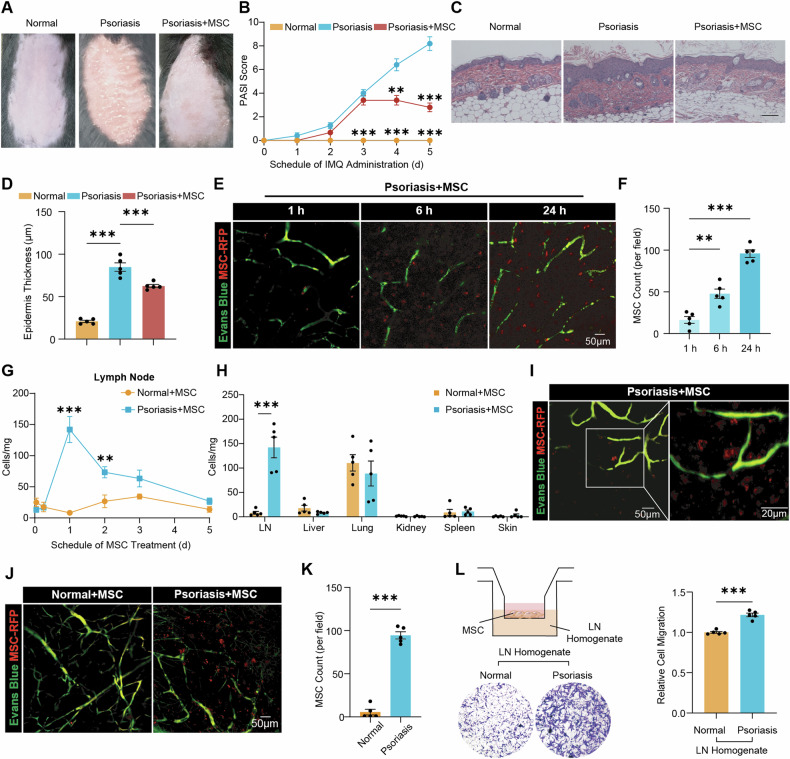
MSC exhibit a homing tendency toward lymph nodes in IMQ-induced psoriatic mice. **A** Photographic documentation illustrating the condition of the mouse back skin five days after MSC treatment. **B** Clinical scoring based on skin erythema, scaling, and thickness, with significant differences compared to those of IMQ-induced psoriatic mice (n = 5). **C** Hematoxylin-eosin (H&E) staining of lesional skin. Scale bar, 100 μm. **D** Quantification of epidermal thickness (n = 5). **E**, **F** IVIM imaging and quantification showing dynamics of transplanted MSC in inguinal lymph nodes from psoriatic mice at 1, 6, and 24 h post-MSC treatment. **G** Dynamic profiling of MSC engraftment in peripheral lymph nodes of normal mice and psoriatic mice assessed using Q-PCR at intervals of 1, 6, 24, 48, 72, and 120 h post-MSC administration, with significant differences compared to normal mice (n = 5). **H** Quantification of MSC in different tissues of normal mice and psoriatic mice assessed 24 h post-administration (n = 5). **I** Localized magnification of MSC in inguinal lymph nodes from psoriatic mice at 24 h post-MSC treatment as determined by IVIM imaging. Scale bars, 50 μm and 20 μm. **J**, **K** Representative images and quantification of RFP-MSC in the inguinal lymph nodes of normal mice and psoriatic mice 24 h after MSC injection. Scale bar, 50 μm. **L** Representative images and quantification of MSC recruited by homogenates of lymph nodes from normal mice and psoriatic mice (n = 5). The data are presented as the means ± SEMs. *P < 0.05, **P < 0.01 and ***P < 0.001. MSC mesenchymal stromal cells, IMQ imiquimod, PASI Psoriasis Area and Severity Index, LN lymph node.

Despite the success of MSC therapy in psoriasis mouse models via topical application [15, 16], MSC are primarily delivered intravenously in clinical trials [5]. To better understand the efficacy and tissue distribution of MSC post-intravenous injection in psoriatic mice, C57BL/6 mice received 5 consecutive days of imiquimod (IMQ) administration. On the first day, 8 × 10^5^ human umbilical cord MSC were administered via the tail vein (Fig. S1A). Disease progression was indicated by increasing Psoriasis Area and Severity Index (PASI) scores (Fig. 1A, B) and body weight loss (Fig. S1B, C) under sustained IMQ application. In contrast, MSC treatment markedly reversed scaling, erythema, and thickening of the back skin (Fig. 1A, B and S1D). IMQ-induced keratinocyte proliferation was effectively alleviated by MSC therapy (Fig. S1E, F).

Next, Q-PCR assays were performed to evaluate the presence of MSC in psoriatic mice after intravenous injection. Most of the cells were cleared from the circulation within fifteen minutes. In both psoriatic and normal mice, MSC were initially trapped in the lung and subsequently redistributed to other organs (Fig. S1G). Remarkably, trace amounts of MSC were detected in the skin (Fig. S1H), suggesting that their therapeutic effects in psoriatic mice may involve targeting other organs. Lymph nodes are essential for autoimmune disease development [17]. Intravital multiphoton (IVIM) imaging revealed the homing of MSC to the lymph nodes. MSC, labeled with red fluorescent protein (RFP), were observed to gradually accumulate within the lymph nodes of psoriatic mice through blood vessels (Fig. 1E, F, Video S1 and S2). Twenty-four hours post treatment, a substantial accumulation of MSC was detected within the lymph nodes. Quantitative polymerase chain reaction (Q-PCR) analysis confirmed the significant migration of transplanted cells to the lymph nodes in psoriatic mice, reaching a peak of 142 cells per milligram of tissue (Fig. 1G). MSC persisted in the lymph nodes up to day five, the treatment endpoint. Compared to other organs, lymph nodes were identified as the primary target site for MSC in psoriatic mice (Fig. 1H). Moreover, MSC maintained an intact morphological structure within the lymph nodes (Fig. 1I and Video S3). Conversely, the engraftment of MSC in the lymph nodes of normal mice was found to be significantly lower compared to that in psoriatic mice (Fig. 1H, J). The area under the curve (AUC) of MSC in lymph nodes of psoriatic mice was 3.32 times greater than in normal mice (Table S1). In both SLE and colitis mouse models, Q-PCR and IVIM imaging consistently corroborated the notable localization of MSC within the lymph nodes (Fig. S1I–M).

In vitro validation confirmed chemotactic responsiveness of MSC toward lymph nodes. When lymph node homogenate of psoriatic mice was added to the lower chambers, there was significant MSC migration, surpassing that observed with lymph node homogenate of normal mouse (Fig. 1L). The analysis of homogenates from mice with SLE and colitis revealed similar trends (Fig S1O and P), indicating a robust tendency of MSC to home toward lymph nodes in the context of autoimmune diseases.

### CCL21-CCR7 axis mediates MSC homing to lymph nodes

**Fig. 2:**
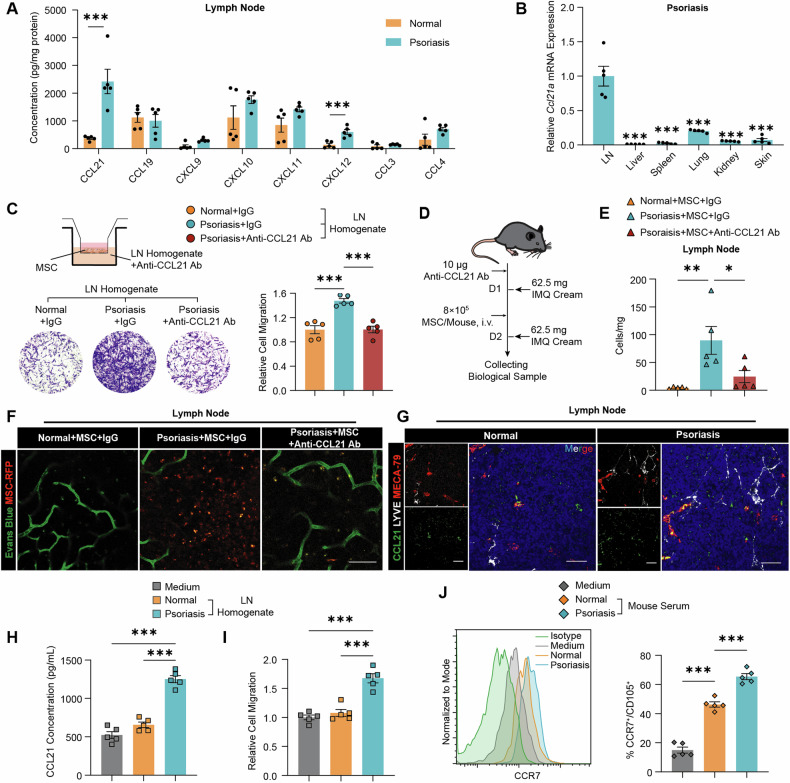
CCL21-CCR7 axis mediates MSC homing to lymph nodes. **A** Measurement of chemokine concentrations in peripheral lymph nodes from psoriatic mice (n = 5). **B** Relative *Ccl21a* expression in different tissues of psoriatic mice (n = 5). Significant differences in comparison with lymph nodes. **C** Representative images and quantification of MSC recruited by the homogenate of lymph node from psoriatic mice in the presence of anti-CCL21 antibody (n = 5). **D** Illustration of the scheme depicting MSC treatment for psoriatic mice pretreated with anti-CCL21 antibody. **E** Quantification of MSC in the peripheral lymph nodes of psoriatic mice pretreated with anti-CCL21 antibody 24 h after MSC administration (n = 5). **F** In vivo IVIM imaging of MSC-RFP in inguinal lymph nodes from psoriatic mice pretreated with anti-CCL21 antibody. Scale bar, 100 μm. **G** Immunofluorescence colocalization analysis of CCL21 with high endothelial cells (MECA-79) or lymphatic endothelial cells (LYVE) in the peripheral lymph nodes of normal mice and psoriatic mice. Scale bars, 100 μm. **H** Secreted protein levels of CCL21 in SVEC-10 cells stimulated with lymph node homogenate for 16 h were analyzed 8 h after media refreshment (n = 5). **I** Quantification of MSC recruited in a transwell culture system by conditioned medium collected after the stimulation of SVEC4-10 cells with lymph node homogenate for 16 h (n = 5). **J** CCR7 expression in MSC stimulated with mouse serum for 24 h was determined by flow cytometry (n = 5). The data are presented as the means ± SEMs. *P < 0.05, **P < 0.01 and ***P < 0.001.

In autoimmune disease models, elevated chemokine levels in lymph nodes attract and direct MSC trafficking [18]. Chemokine profiling has identified C-C motif chemokine ligand 21 (CCL21) as the predominant chemokine in the lymph nodes of psoriatic mice, exhibiting significantly elevated levels subsequent to the administration of IMQ (Fig. 2A). Psoriatic mice also had higher serum CCL21 concentrations than normal mice (Fig. S2A). Similarly, mice with SLE and colitis exhibited significantly elevated levels of CCL21 in the lymph nodes and serum, compared to normal mice (Fig S2B and C). In psoriatic mice, CCL21 exhibited the highest gene (Fig. 2B) and protein expression (Fig. S2D) in the lymph nodes compared to other organs, indicating its specific expression within the lymph nodes. To study the role of CCL21 in MSC migration to lymph nodes, a neutralizing antibody against CCL21 was added to the homogenates of lymph nodes from psoriatic mice, reducing MSC migration in a transwell culture system (Fig. 2C). In vivo, pre-injection of the anti-CCL21 antibody reduced MSC migration to lymph nodes by 72.6% (Fig. 2D, E). CCL21 depletion altered the distribution of MSC in psoriatic mice (Fig. S2E). IVIM imaging of the inguinal lymph nodes supported these findings (Figs. 2F and S2F).

Subsequently, an analysis identified the cellular sources of CCL21 within the lymph nodes [19]. Single-cell sequencing (scRNA-seq) revealed that CCL21 was one of the most highly expressed chemokines (Fig. S3A–C), with the highest levels in high endothelial cells (HECs) and T-cell zone reticular cells (TRCs, Fig. S3D). Immunofluorescence staining confirmed CCL21 expression in HECs and TRCs within the lymph nodes of psoriatic mice (Fig. 2G and Fig. S3E–G). CCL21 was significantly more expressed in HECs compared to lymphatic endothelial cells and was higher in psoriatic mice compared to normal mice (Fig. S3E). However, no significant difference in CCL21 production by stromal cells was detected between the two groups (Fig. S3G). These findings suggest that the heightened expression of CCL21 in HECs within the context of inflammatory conditions may promote MSC migration toward lymph nodes.

To validate the influence of inflammation on the CCL21-CCR7 axis, lymph node vascular endothelial cells (SVEC4-10) were stimulated with homogenates of lymph nodes from psoriatic mice. This led to a significant increase in CCL21 secretion, which was absent in the lymph nodes of normal mice (Fig. 2H and S3H, I). The conditioned medium from stimulated SVEC4-10 cells caused a greater migration of MSC (Fig. 2I). Additionally, C-C motif chemokine receptor 7 (CCR7) expression on MSC was significantly upregulated after exposure to serum from psoriatic mice, both in fluorescence intensity and proportion of CCR7-positive cells (Figs. 2J and S3J, K). This upregulation was greater than that in normal mice, indicating the activation of the CCL21-CCR7 axis in psoriatic mice.

### MSC treatment restrains the Th17 cell response and dampens inflammation

**Fig. 3:**
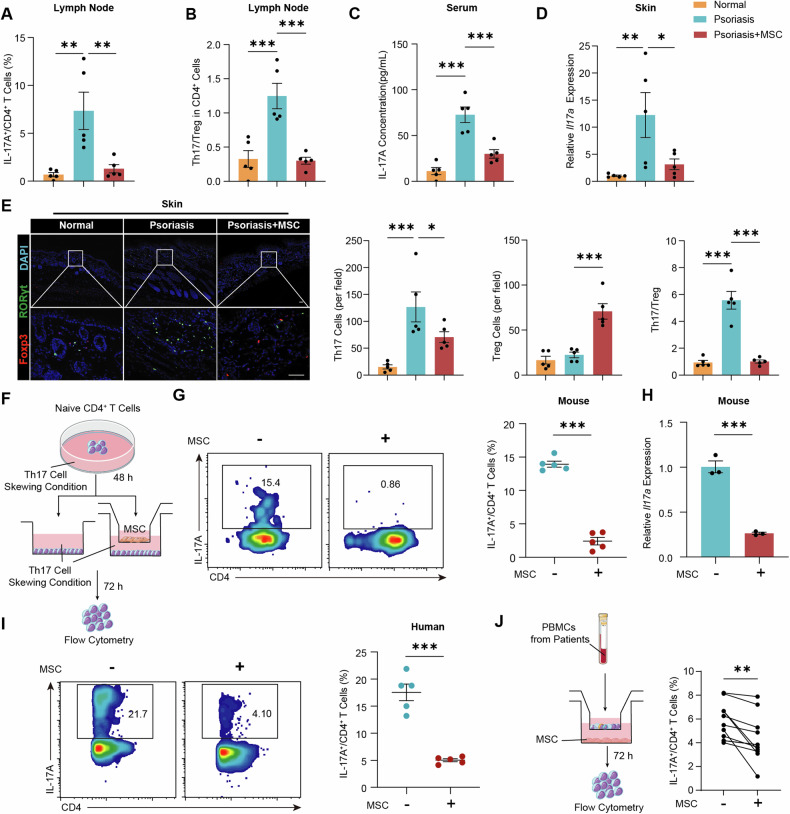
MSC treatment restrains the Th17 cell response and dampens inflammation. **A** Populations of Th17 cells in the peripheral lymph nodes of psoriatic mice quantified using flow cytometry 5 days after MSC treatment (n = 5). **B** The ratio of Th17 cells to Treg cells in the peripheral lymph nodes (n = 5). **C** Serum levels of IL-17A (n = 5). **D** *Il17a* expression in the psoriatic skin of mice (n = 5). **E** Representative immunofluorescence images and quantification of infiltrated Th17 cells and Treg cells in lesional skin tissue sections (n = 5). Scale bar, 50 μm. **F**–**I** Naïve CD4^+^ T cells, activated under Th17 cell polarizing condition for 48 h, were subsequently co-cultured with MSC at a ratio of 50:1 in a transwell culture system for an additional 3 days. **F** Experimental scheme detailing T cells co-cultured with MSC. **G** Flow cytometry analysis of the frequency of Th17 cells after the co-culture of murine naïve CD4^+^ T cells isolated from mouse spleen with MSC under Th17 cell polarizing condition (n = 5). **H** *Il17a* expression in murine T cells after co-cultured with MSC under Th17 cell polarizing condition (n = 3). **I** Frequency of Th17 cells after the co-culture of human naïve CD4^+^ T cells isolated from healthy donors with MSC under Th17 cell polarizing condition (n = 5). **J** Fresh PBMCs derived from psoriatic patients were co-cultured with MSC at a ratio of 50:1 employing a transwell culture system, and the proportion of Th17 cells was analyzed after 72 h. Each line represents an independent biological sample (n = 11). The data are presented as the means ± SEMs. *P < 0.05, **P < 0.01 and ***P < 0.001. DC dendritic cell, NC negative control, PBMC peripheral blood mononuclear cell.

The regulatory effects of MSC on immune cells within the lymph nodes were explored following IMQ modeling. A notable increase in the number of Th1 cells (Fig. S4A, B), Th17 cells (Fig. 3A and Fig S4C), and mature DCs (Fig. S4D) was observed after IMQ administration. The proportions of Th1 cells and mature DCs decreased slightly, while the proportion of Treg cells mildly increased following MSC treatment (Fig. S4E). The percentage of Th17 cells reduced from 7.3% to 1.3% in the lymph nodes, with a corresponding decrease in RORγt expression. The normalization of the Th17/Treg cell ratio indicated a reversal of the immune imbalance induced by IMQ (Fig. 3B). Consistent with these cellular changes, a significant reduction in the serum IL-17A and IFN-γ concentrations was observed (Fig. 3C and Fig. S4F). The expression levels of inflammatory factors in the lesional skin were markedly reduced (Fig. 3D and Fig. S4G). The infiltration of Th17 cells into the skin diminished, whereas the population of Treg cells augmented, collectively contributing to the restoration of immune homeostasis (Fig. 3E). These findings were also confirmed in SLE and colitis mouse models, in which MSC treatment also led to a reduction in the Th17 cell response (Fig. S4H–L).

The inhibitory effect of MSC on Th17 cells was further confirmed in vitro. Murine naïve CD4^+^ T cells were activated under Th17 cell polarizing conditions for 48 h and then co-cultured with MSC in a transwell culture system for an additional 3 days (Fig. 3F). Flow cytometry revealed a 79.6% reduction in the proportion of Th17 cells (Fig. 3G). *Il17a* gene expression decreased 3.82-fold in murine T cells following co-culture with MSC (Fig. 3H). The regulatory effects of MSC on Th17 cells were also validated with human lymphocytes from healthy donors under Th17 cell polarizing conditions (Fig. 3I). MSC significantly decreased the percentage of IL-17A^+^ cells from 17.5% to 5.0%. To validate these observations in a clinical context, peripheral blood mononuclear cells (PBMCs) from psoriatic patients were co-cultured with MSC (Fig. 3J). A significant decrease in the percent of Th17 cells was observed after incubation, confirming the modulatory impact of MSC on Th17 cells. MSC were primarily localized in the T-cell zone of lymph nodes post-migration (Fig. S4N, O). In both psoriatic and SLE mice, MSC were found to be in close spatial proximity to CD4^+^ T cells, suggesting direct regulation of Th17 cells by MSC.

### MSC secrete LAAO in response to inflammation through the NF-κB pathway

**Fig. 4:**
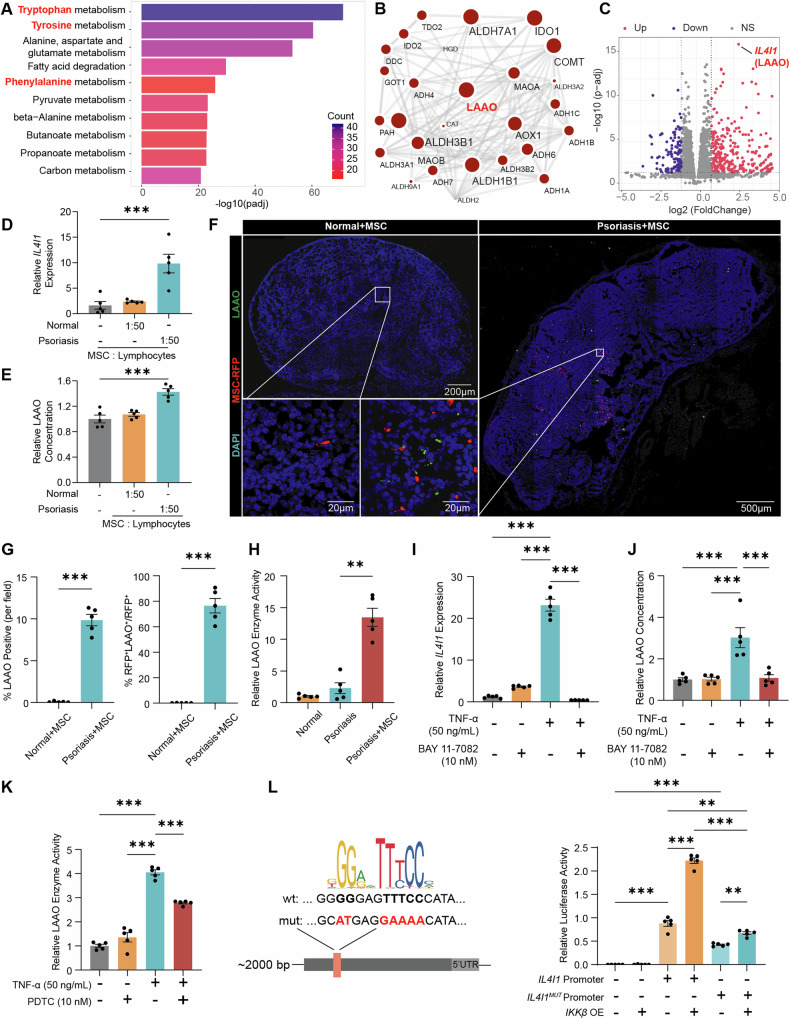
MSC secrete LAAO in response to inflammation through the NF-κB pathway. Transcriptome analysis of MSC co-cultured with PBMCs isolated from psoriatic patients for 48 h (n = 5). **A** KEGG enrichment analysis of DEGs after incubation. **B** Protein-protein interaction network of DEGs based on the STRING online database. The node size indicates the adjusted *P*-value of the DEGs, and the edge width indicates the combined score. **C** Volcano plot illustrating DEGs. **D** Assessment of *IL4I1* expression in MSC co-cultured with murine lymphocytes at a ratio of 1:50 (n = 5). **E** Quantification of LAAO secretion in MSC co-cultured with murine lymphocytes at a ratio of 1:50 (n = 5). **F** Immunofluorescence colocalization analysis of human LAAO with MSC in the lymph nodes of psoriatic mice and normal mice 24 h after MSC infusion. Scale bars, 500 μm, 200 μm and 20 μm. **G** Quantification of LAAO immunofluorescence and colocalization of LAAO with MSC using CellProfiler in the lymph nodes of psoriatic mice (n = 5). **H** Measurement of LAAO enzyme activity in the lymph nodes of psoriatic mice 24 h after MSC infusion (n = 5). **I** *IL4I1* gene expression in MSC after treatment with TNF-α for 4 h, along with the NF-κB inhibitor BAY 11-7082, which antagonizes the phosphorylation of IκBα (n = 5). **J** Protein levels of LAAO in the lysate of MSC after treatment with TNF-α for 24 h, in addition to BAY 11-7082 (n = 5). **K** LAAO enzyme activity in the lysate of MSC after treatment with TNF-α for 24 h, along with PDTC, an inhibitor of IκB ubiquitin ligase activity (n = 5). **L** NFKB1 binding sites in the human *IL4I1* promoter region predicted by JASPAR. The luciferase activity of the wild-type and mutant *IL4I1* promoters in HEK293T cells after *IKKβ* overexpression. The data are presented as the means ± SEMs. *P < 0.05, **P < 0.01 and ***P < 0.001. LAAO L-amino-acid oxidase, NS not significant.

To understand how MSC regulate immune responses, RNA-seq analysis was performed on MSC incubated with PBMCs from psoriatic patients (Fig. S5A). KEGG enrichment analysis revealed significant changes in phenylalanine, tryptophan, and tyrosine metabolism (Fig. 4A), implying the potential involvement of LAAO in the regulatory interaction between MSC and Th17 cells in autoimmune responses (Fig. 4B-C and Fig. S5B). Exposure of MSC to splenic lymphocytes from psoriatic mice increased *IL4I1* (gene name of LAAO) expression (Fig. 4D) and LAAO secretion in MSC (Fig. 4E). In contrast, LAAO production was not induced by the lymphocytes of normal mice (Fig. 4D, E). Immunofluorescence analysis revealed increased expression of LAAO and a higher ratio of LAAO^+^ MSCs in the lymph nodes of mice with autoimmune diseases, whereas minimal LAAO expression was observed in normal mice (Fig. 4F, G and S5C, D). LAAO enzyme activity in the lymph nodes yielded compelling results (Fig. 4H), suggesting the production of LAAO by MSC is linked to the inflammatory environment, emphasizing the context-dependent immunomodulatory effects of MSC [20].

We investigated how the inflammatory microenvironment regulates LAAO production. Tumor necrosis factor-α (TNF-α) levels were significantly increased in mice with autoimmune diseases (Fig. S5E). Further research showed that TNF-α upregulated *IL4I1* mRNA levels in MSC over time (Fig. S5F). Blocking the NF-κB pathway reversed this effect (Fig. 4I). TNF-α also increased LAAO production and secretion, as well as enzyme activity (Figs. 4J, K and S5G, H). We conducted a dual luciferase assay using the *IL4I1*-promoter-PGL4.10 reporter plasmid. NFKB1 binding sites in the *IL4I1* promoter region were identified with the JASPAR database, and a mutant promoter plasmid was generated. Overexpression of *IKKβ* increased *IL4I1* promoter activity by 2.5-fold (Fig. 4L). The mutant promoter showed a lower increase, suggesting TNF-α induces LAAO production in MSC through the NF-κB pathway.

### LAAO-dependent suppression of Th17 cells by MSC

**Fig. 5:**
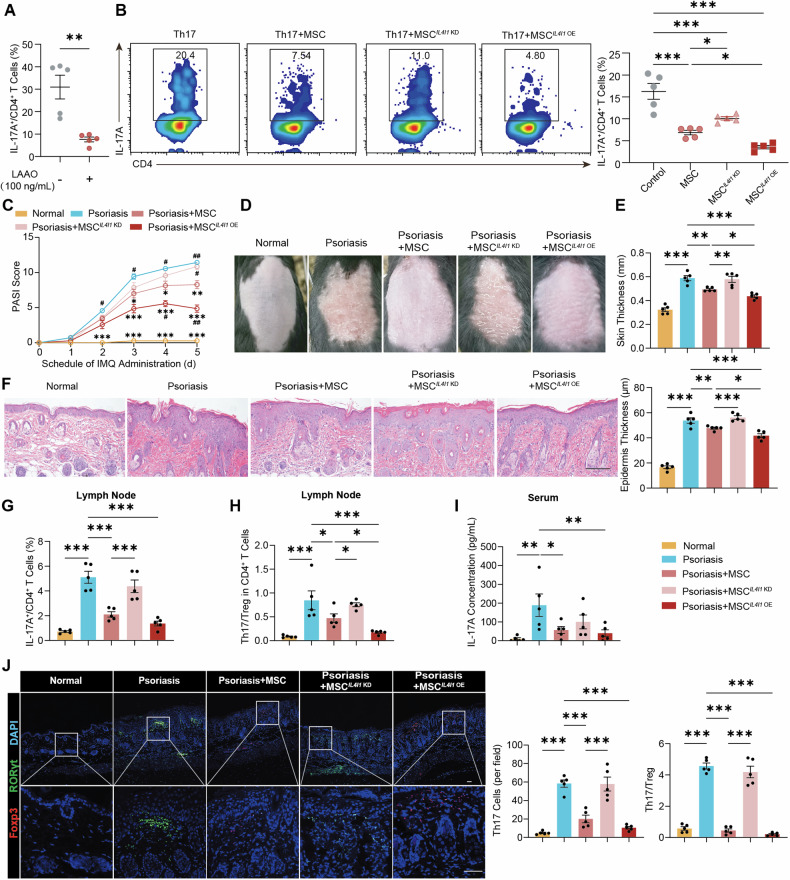
LAAO-dependent suppression of Th17 cells by MSC. **A** Flow cytometry analysis of the percent of IL-17A^+^ cells among murine CD4^+^ T cells differentiated under Th17 cell polarizing condition containing recombinant LAAO protein (n = 5). **B** Flow cytometry analysis of the frequency of Th17 cells after the co-culture of murine naïve CD4^+^ T cells under Th17 cell polarizing condition with MSC featuring *IL4I1* knockdown (MSC^*IL4I1* KD^) or overexpression (MSC^*IL4I1* OE^) at a ratio of 50:1, using a transwell culture system for an additional 3 days (n = 5). **C**–**J** Psoriatic mice were intravenously injected with 8 × 10^5^ MSC featuring *IL4I1* knockdown or overexpression. **C** Clinical scores were monitored until day five (n = 5). Comparisons with psoriatic mice are represented by *, and comparisons with psoriatic mice treated with wild-type MSC are represented by #. **D** Photographic documentation illustrating the condition of mouse back skin on day five. **E** Measurement of thickness of the mouse back skin on day five (n = 5). **F** H&E staining of lesional skin and quantification of epidermal thickness (n = 5). Scale bar, 100 μm. **G** Proportion of Th17 cells in the peripheral lymph nodes (n = 5). **H** The ratio of Th17 cells to Treg cells in the peripheral lymph nodes (n = 5). **I** Serum levels of IL-17A (n = 5). **J** Representative immunofluorescence images and the quantification of infiltrated Th17 and Treg cells in lesional skin tissue sections (n = 5). Scale bar, 50 μm. The data are presented as the means ± SEMs. *P < 0.05, **P < 0.01 and ***P < 0.001. ^#^*P* < 0.05, ^##^*P* < 0.01 and ^###^*P* < 0.001.

To clarify the regulatory effect of MSC-derived LAAO on Th17 cells, recombinant human LAAO protein (Fig. 5A) or supernatants from cells overexpressing human *IL4I1* (Fig. S6A) were added to the Th17 cell differentiation medium. After a 3-day incubation, flow cytometry showed a notable inhibitory effect of LAAO on Th17 cells. To systematically assess the involvement of MSC-derived LAAO in the immunomodulatory effects of MSC, we engineered stable *IL4I1* knockdown (MSC^*IL4I1* KD^) or overexpressing (MSC^*IL4I1* OE^) MSC via lentiviral transfection (Fig. S6B–D). MSC^*IL4I1* KD^ exhibited a mild inhibitory effect on Th17 cells, that was less pronounced than that of wild-type MSC (Fig. 5B and Fig. S6E). Conversely, MSC^*IL4I1* OE^ significantly inhibited Th17 cells to a greater extent than wild-type MSC.

For in vivo validation, psoriatic mice treated with wild-type MSC, MSC^*IL4I1* KD^, or MSC^*IL4I1* OE^ were sacrificed 5 days post injection (Fig. S6F). Evaluation of PASI scores (Fig. 5C) and the condition of the back skin (Figs. 5D–F and S6G-H) revealed a notable reduction in MSC efficacy following *IL4I1* knockdown, while *IL4I1* overexpression significantly enhanced MSC efficacy. After treatment with MSC^*IL4I1* KD^, scaling, erythema, and thickening of the back skin remained severe. In contrast, compared with treatment with wild-type MSC, treatment with MSC^*IL4I1* OE^ led to a more efficient decrease in Ki-67 expression. The proportion of Th17 cells in the lymph nodes and lesional skin was higher in mice treated with MSC^*IL4I1* KD^ than in those treated with wild-type MSC (Fig. 5G-J). Additionally, the modulation of the immune microenvironment by MSC infusion was diminished after *IL4I1* knockdown (Fig. S6I–K). Conversely, MSC^*IL4I1* OE^ exhibited a stronger ability to regulate the Th17/Treg cell ratio in both lymph nodes and lesional skin, indicating that the therapeutic effect of MSC^*IL4I1* OE^ is superior to that of wild-type MSC. Thus, these results suggest that LAAO plays a vital role in the ability of MSC to suppress Th17 cells, emphasizing its potential as a key therapeutic factor in autoimmune disorders.

### Indole-3-pyruvic acid is a key metabolite that mediates LAAO-induced Th17 cell inhibition

**Fig. 6:**
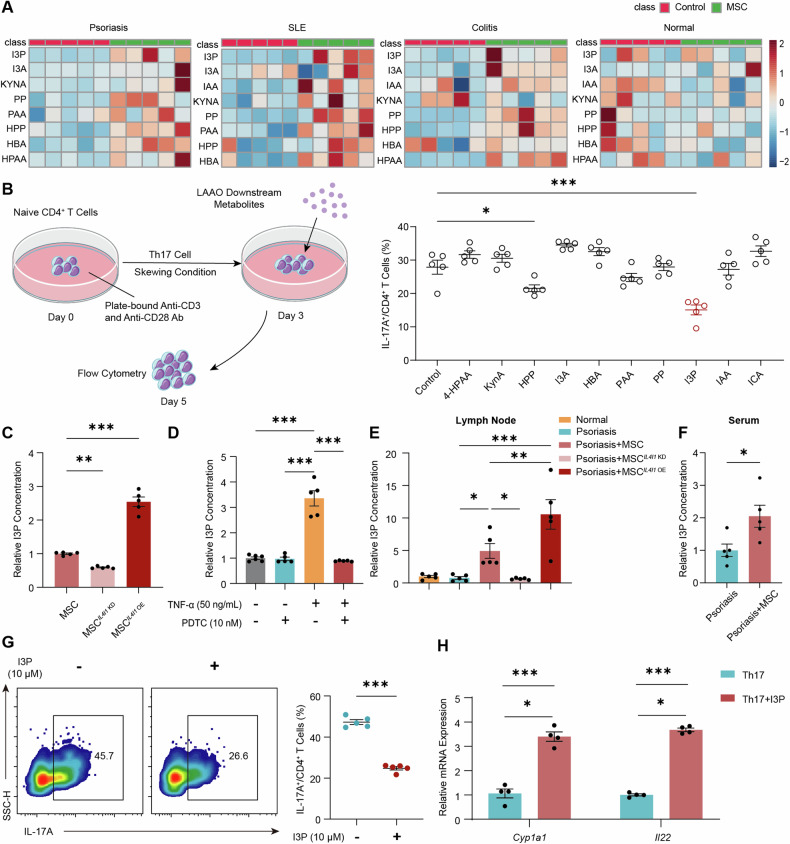
Indole-3-pyruvic acid is a key metabolite that mediates LAAO-induced Th17 cell inhibition. **A** Differential analysis of LAAO downstream metabolite concentrations in lymph nodes from mice 5 days after MSC infusion (n = 5). **B** Human CD4^+^ T cells from healthy donors differentiated under Th17 cell polarizing condition and treated with LAAO downstream metabolites (10 μM) for 48 h. Flow cytometry analysis of cytokine staining in human Th17 cells (n = 5). **C** Secreted I3P concentration in MSC featuring *IL4I1* knockdown or overexpression (n = 5). **D** Secreted I3P concentration in MSC treated with TNF-α for 24 h in addition to PDTC (n = 5). **E** I3P concentration in the lymph nodes of psoriatic mice treated with MSC featuring *IL4I1* knockdown or overexpression (n = 5). **F** I3P concentration in the serum of psoriatic mice treated with MSC after 5 days (n = 5). **G** Murine CD4^+^ T cells differentiated under Th17 cell polarizing condition and treated with I3P (10 μM) for 48 h. Flow cytometry analysis of cytokine staining in murine Th17 cells. Representative plots and quantification of Th17 cells (n = 5). **H** Q-PCR analysis of the relative expression of AHR downstream genes in murine CD4^+^ T cells differentiated under Th17 cell polarizing condition treated with I3P (10 μM) for 48 h (n = 4). The data are presented as the means ± SEMs. *P < 0.05, **P < 0.01 and ***P < 0.001. I3A Indole-3-aldehyde, I3C Indole-3-carboxaldehyde, I3P Indole-3-pyruvic acid, IAA Indole-3-acetic acid, HBA 4--hydroxybenzaldehyde, HPP 4-hydroxyphenylpyruvic acid, HPAA 4-hydroxyphenylacetic acid, KYNA kynurenic acid, PAA phenylacetic acid, PP phenyl pyruvic acid.

LAAO exhibits a distinct preference for L-amino acids, converting phenylalanine, tyrosine, and tryptophan into phenyl pyruvic acid, hydroxyphenyl pyruvic acid, and I3P, respectively [21]. To quantitatively assess the concentrations of downstream metabolites of LAAO, we conducted UPLC-MS/MS analysis of lymph nodes from psoriatic, SLE, colitis, and normal mice following MSC infusion. A significant increase in downstream metabolite levels within the lymph nodes was observed after MSC treatment (Fig. 6A), likely due to the specific distribution of MSC in the lymph nodes.

We hypothesize that among these metabolites, some may hinder Th17 cell function [22, 23]. Human naïve CD4^+^ T cells from healthy donors were exposed to these metabolites under Th17 cell polarizing conditions (Fig. 6B). Among the 10 metabolites examined, I3P emerged as the most potent inhibitor of Th17 cell differentiation. We further confirmed the capacity of MSC-derived LAAO to generate I3P. *IL4I1* overexpression in MSC increased I3P secretion, while *IL4I1* knockdown led to decreased secretion (Fig. 6C). TNF-α increased I3P production in MSC by 3.35-fold, an effect largely reversed by the NF-κB inhibitor (Fig. 6D). Additionally, *IL4I1* overexpression elevated I3P levels in lymph nodes from psoriatic mice after MSC transplantation, whereas MSC^*IL4I1* KD^ failed to increase I3P level (Fig. 6E), potentially explaining their inability to inhibit Th17 cell function. I3P concentration in the serum of psoriatic mice also increased after MSC administration (Fig. 6F). The regulatory impact of I3P on Th17 cell differentiation was further validated using murine naïve CD4^+^ T cells (Fig. 6G). The fraction of IL-17A^+^ cells decreased from 47.3% to 25.7% with the addition of I3P, and AHR pathway genes like *Cyp1a1* and *Il22* were upregulated, indicating AHR pathway activation by I3P in Th17 cells (Fig. 6H). Overall, these findings indicate that LAAO derived from MSC regulates Th17 cell function through I3P.

## Disscussion

In this study, we investigated the role of MSC in suppressing Th17 cells within lymph nodes via LAAO secretion. Despite promising outcomes in clinical trials [24], understanding their therapeutic mechanism remains challenging. A critical consideration is whether enough MSC can reach the injury sites and persist for an adequate duration. Our findings indicate that MSC are primarily trapped in the lungs, with only a minimal fraction reaching the skin in psoriatic mice, despite their efficacy. Intriguingly, MSC exert their therapeutic effect by targeting the lymph nodes, a central immune hub. This achievement is often beyond the reach of conventional chemical drugs. Unlike the persistent and severe adverse effects linked to continuous administration of immunosuppressive agents, MSC offer a safer alternative with prolonged efficacy after a single treatment [25–27]. Our research revealed that MSC persisted in the lymph nodes for more than 5 days, providing sustained immunoregulatory efficacy. Previous studies lacked assessments of distribution pattern of the migrated MSC in mice with autoimmune diseases [28–30]. In this study, we addressed this gap by quantifying the MSC exposure using Q-PCR. Furthermore, the migration of MSC to the lymph nodes was visualized through IVIM imaging and immunofluorescence techniques.

Chemotactic factors drive MSC toward injury sites to alleviate inflammation [31, 32]. CXCL12 elevation at the injury sites is critical for this step [33, 34]. However, lymph node showed different chemokine secretion pattern [35, 36]. Therefore, the specific homing of MSC to the lymph nodes in mice with autoimmune diseases may be guided by other chemokines. Unlike CXCL12, which is widely expressed, CCL21 is specifically produced in lymph nodes and has a highly specific and efficient chemotactic effect on lymphocytes [37]. We found HECs and TRCs secrete CCL21, guiding MSC to T-cell zones within lymph nodes along a concentration gradient. MSC overexpressing CCR7 migrated more effectively to the spleen and lymph nodes in a GvHD model [38]. The disease state enhances MSC distribution in lymph nodes, with inflammation increasing the CCL21 concentration and MSC surface CCR7 expression. This finding aligns with the genome-wide association study linking CCL21/CCR7 expression to disease severity in SLE patients [37]. Although CCL19 is another CCR7 ligand, neutralizing CCL21 substantially reduces the homing of MSC to lymph nodes, indicating the critical role of CCL21. Depleting CCL21 in psoriatic mice results in the distribution pattern of the migrated MSC similar to that in normal mice. Our research provides novel insights into the molecular mechanism of MSC homing.

MSC are known to modulate the balance between Th17 and Treg cells via IDO and TGF-β [39]. Our research revealed that MSC directly inhibit Th17 cell response in vitro and in vivo, independent of Treg regulation. Although IL-17A and IL-17 receptor antibodies benefit psoriatic patients [40, 41], they failed to treat active Crohn’s disease [42]. Autoimmune disorders involve various immune cells and pathways [43–45], challenging for a single-target approach. MSC therapy regulates diverse immune cells within lymph nodes and restores immune homeostasis. Our study concentrated on the impact of MSC on Th17 cells, but further investigation into their effects on other immune cell types is warranted.

The inhibitory effect on Th17 cells is attributed to LAAO, which has been reported to inhibit T and B cell proliferation [46]. Recent studies highlight LAAO as an independent metabolic immune checkpoint in the tumor microenvironment, which is distinct from IDO [21]. Our study demonstrated that LAAO ablation substantially reduced the inhibitory effect of MSC on Th17 cells, emphasizing the importance of LAAO in MSC-mediated immunomodulation. This finding complements the known role of IDO in immune regulation [39]. Unlike intracellular enzymes such as IDO, secreted LAAO can impact a wider range via blood and lymphatic circulation for immunomodulation [47]. Our research confirmed increased downstream metabolites of LAAO in lymph nodes after MSC treatment, accompanied by elevated serum I3P levels. Despite the limited quantity and duration of transplanted MSC, MSC-derived LAAO can enter the extracellular matrix through paracrine secretion, leading to the generation of numerous metabolites influencing more cells over broader spatial and temporal range, potentially explaining the long-term efficacy of single MSC treatment.

LAAO preferentially catalyzes the reactions of phenylalanine, tyrosine, and tryptophan. Beyond its enzymatic role, LAAO is associated with immune regulatory functions involving amino acid depletion and H_2_O_2_ formation [48]. Increasing LAAO expression enhances TNF-stimulated gene 6 synthesis (TSG-6) in muscle stem cells, contributing to immunosuppressive effects on neutrophils [49]. Recent findings highlight the role of LAAO in tumor progression via indole metabolite generation [21]. Our research revealed that LAAO-mediated I3P limited the Th17 cell response. AHR is crucial in immune and inflammatory processes. I3P activates AHR at lower concentrations than kynurenine or kynurenic acid [21]. Indole metabolites including I3P were historically thought to be produced by microbial [50, 51]. Our data suggested that MSC-derived LAAO is a new source of I3P, inhibiting Th17 cell function via the AHR pathway, potentially explaining the immunomodulatory effects of MSC. Other LAAO-derived metabolites may also regulate immune cells and merit further investigation.

A significant challenge in the clinical application of MSC is their variable efficacy among patients, leading to unpredictable outcomes [2]. Our research emphasizes that inflammation significantly impacts both the distribution and efficacy of MSC. While MSC are conventionally believed to home to the injury sites, our investigation revealed an additional tendency for lymph node targeting in autoimmune disease models, which is absent in normal mice. Moreover, the secretion of immunomodulatory factors depends on the level of inflammation. Clinical trials have demonstrated that serum IFN-γ levels serve as a predictive indicator for the therapeutic efficacy of MSC transplantation in SLE patients [52]. This association is attributed to the ability of IFN-γ to upregulate IDO expression in MSC. Our research further suggested that serum CCL21 and TNF-α concentration should also be considered as an important indicator, as they are directly correlated with the homing efficiency of MSC and subsequent secretion of LAAO. Therefore, personalized considerations based on the inflammatory status will be crucial for determining the optimal timing and dosage of MSC therapy in clinical settings.

**Fig. 7:**
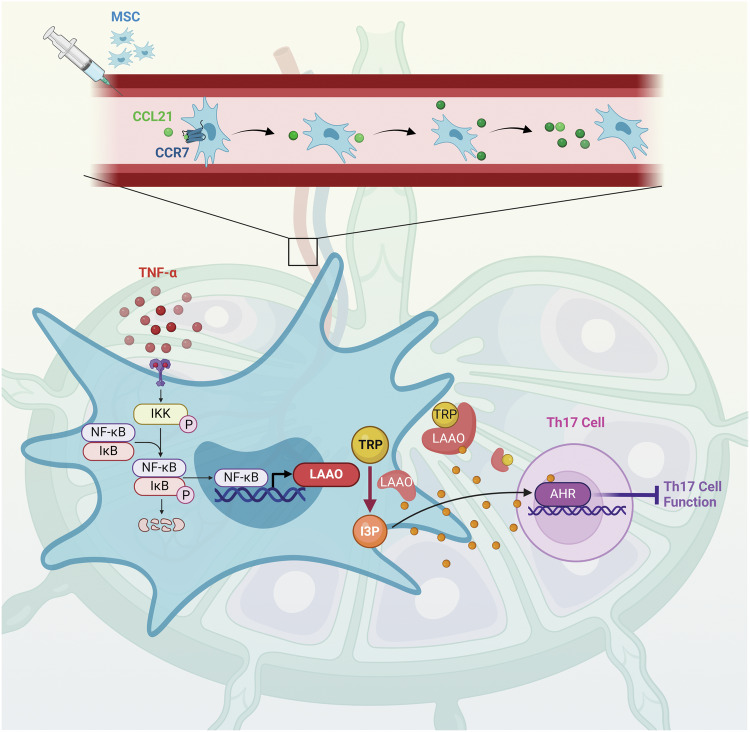
MSC modulate Th17 cell response through LAAO in the lymph nodes via NF-κB activation. In mice with autoimmune diseases, elevated CCL21 levels drive the specific homing of MSC to lymph nodes following transplantation. Within the lymph nodes, MSC respond to TNF-α stimulation by secreting LAAO via NF-κB activation. LAAO catalyzes the production of I3P from tryptophan, which exhibits strong inhibitory effects on Th17 cells by activating the AHR pathway. The figure was created with BioRender (https://biorender.com).

Our study revealed a distinct homing pattern of MSC to lymph nodes in mice with autoimmune diseases, driven by CCL21/CCR7 axis. Within the lymph nodes, MSC effectively inhibit Th17 cells, contributing to the restoration of immune balance in mice. LAAO secretion, triggered by the elevated TNF-α via the NF-κB pathway, is essential in this process, generating I3P to activate the AHR pathway (Fig. 7). These discoveries augment our understanding of the immunomodulatory impacts of MSC in autoimmune disorders, potentially stimulating additional investigation into innovative therapeutic avenues.

## Materials and methods

### Cell culture

Human umbilical cord MSC were generously provided by Jiangsu Renocell Biotech Co., Ltd (Nanjing, China) and verified using cell surface markers, including CD105(+), CD73(+), CD29(+), CD90(+), CD44(+), CD45(−), CD31(−), CD34(−), and HLA-DR (−). The cells were cultured in DMEM/F-12 (Gibco, Wilmington, MA) supplemented with 10% FBS (Gibco). The experimental use of MSC was restricted to passages 3 to 6. SVEC4-10 cells, which are endothelial cells isolated from the vascular epithelium of an adult male mouse, were obtained from Procell (Wuhan, China) and cultured in DMEM supplemented with 10% FBS. All cell were maintained at 37 °C in a humidified incubator with 5% CO_2_.

### Mice

All animal care and experimental procedures followed the National Research Council’s Guidelines for the Care and Use of Laboratory Animals and were approved by the SPF Animal Laboratory of China Pharmaceutical University (Approval Number: 2021-06-023). C57BL/6 (6–8 weeks old, 18–22 g) and MRL/lpr mice from Beijing Vital River Laboratory Animal Technology Co. were documented in a temperature-controlled environment (23 ± 2 °C) with a 12-h light-dark cycle. Sample size was determined using the resource equation approach, with efforts to minimize animal distress and ensure judicious use. Those handling the animals and conducting experiments were blinded to the allocation sequence and group assignment. Randomization determined animal allocation for the experiments.

To induce skin lesions, 6- to 8-week-old female C57BL/6 mice received topical treatment with 62.5 mg of 5% IMQ cream (Aldara, 3M Pharmaceuticals, Stockholm, Sweden) once daily for 5 consecutive days. On day one, 8 × 10^5^ MSC were injected intravenously. To neutralizing CCL21, mice received 10 μg of anti-mouse CCL21 antibody or control IgG (R&D System, Minneapolis, MN) 16 h before MSC infusion. The severity of skin lesions was assessed daily using the PASI score, ranging from 0 (no symptoms) to 4 (severe symptoms) for erythema, scaling, and thickness, and combined for a total score. Five days post-IMQ treatment, major organs, skin, and lymph nodes were collected for analysis, and back skin samples were fixed and stained with H&E and Ki-67. To establish a colitis mouse model, 6- to 8-week-old male C57BL/6 mice were fed with 2.5% dextran sodium sulfate polymers (DSS, 36,000–50,000 MW, MP Biomedicals, Santa Ana, CA) in drinking water for five days, followed by normal drinking water. On day seven, each mouse received 2 × 10^6^ MSC via intraperitoneal injection. Mice were monitored daily for weight loss, rectal bleeding, and diarrhea to track disease progression. On day ten, the mice were euthanized for further analysis. When MRL/lpr mice reached around 16 weeks of age, they were grouped based on a consistent increase in the urinary protein/creatinine ratio and noticeable hair loss. After classification, the mice were intravenously infused with 8 × 10^5^ MSC through the tail vein. Seven days post-treatment, serum and peripheral lymph nodes were collected for further analysis.

### Imaging of MSC in vivo

To visualize MSC homing within the lymph nodes, intravital two-photon microscopy platform IVM-CMS3 (IVIM Technology, Seoul, Korea) was used. MSC were labeled with RFP using a lentiviral vector (Genomeditech, Shanghai, China). To enhance imaging contrast, Evans blue angiography agent or anti-mouse CD31 antibody (IVIM Technology) was injected via the tail vein. Once the angiography agent diffused, the mice were anesthetized. The inguinal lymph node was surgically exposed and imaged using a two-photon microscope equipped with a water objective lens (20×). Imaging was performed in the RFP (570-620 nm, for MSC) and near-infrared (NIR) (619–676 nm, for Evans blue or CD31 antibody) channels.

### Q-PCR analysis

For pharmacokinetic investigations, blood, liver, spleen, lung, kidney, peripheral lymph node, and skin samples were collected at 1, 6, 24, 48, 72, and 120 h post-MSC administration. Genomic DNA was extracted from blood and tissues using the FastPure Cell/Tissue DNA Isolation Mini Kit (Vazyme, Nanjing, China). MSC were quantified using the human-specific *Alu* gene, with quantification calibrated against a standard curve made from the logarithm of cell numbers and their corresponding CT values.

To assess *IL4I1* expression, MSC were stimulated with 50 ng/mL TNF-α (Peprotech, Rocky Hill, NJ). To suppress the NF-κB pathway, 10 μM BAY 11-7082 (MedChemExpress, Monmouth Junction, NJ) was added 2 h before TNF-α stimulation. Total mRNA was extracted from tissues and cells using RNAiso Plus (TAKARA, Kusatsu, Japan), and cDNA synthesis was performed with HiScript II Q Select RT SuperMix (Vazyme). Q-PCR was carried out with iTaq™ Universal SYBR® Green SuperMix (Vazyme) on a CFX96 real-time PCR detection system (Bio-Rad, Hercules, CA). The primers for Q-PCR are listed in Table S2.

### Transwell migration assay

Transwell inserts (8.0 μm pore size, Corning, Glendale, AZ) were placed in 24-well culture plates. MSC were added to the upper chambers in 200 μL of DMEM/F12, while the lower chambers contained complete medium with tissue homogenate from mouse lymph nodes, anti-mouse CCL21 antibody or IgG (1 μg/mL, R&D System), and medium supernatant collected from SVEC4-10 cell stimulation with lymph node homogenate. After a 24 h incubation, nonmigratory cells in the upper chamber were removed, and those on the underside were fixed, stained with crystal violet. Cell migration was quantified in five randomly selected microscope fields using ImageJ software.

### scRNA-seq data analysis

To analyze the scRNA-seq data, we used a data matrix of non-hematopoietic cells in mouse lymph nodes (GSM5819066) from the GEO database (https://www.ncbi.nlm.nih.gov/gds/). Skin-draining lymph nodes (inguinal and axillary) were resected, digested, and sorted for scRNA-seq. We processed the gene expression matrix with Cell Ranger (v6.0.0) and used Seurat (v4.2.0) for normalization, dimensionality reduction, and cell clustering. We excluded cells with fewer than 750 gene counts or more than 4500 gene counts, and those with mitochondrial gene percentages exceeding 6%. Principal component analysis was used for dimensionality reduction, selecting the first 10 principal components for cluster generation. Clusters were defined using both the K-means algorithm and a graph-based approach. Cell types were identified based on known cell markers from previous literature [19, 52–54].

### Human and murine T-cell differentiation

Human blood samples were obtained with informed consent and approved by the Human Ethics Committee of the Jiangsu Province Hospital of Chinese Medicine (ID: 2017NL-056-02). PBMCs were isolated by density gradient centrifugation using Ficoll (GE, Marlborough, MA). Naïve CD4^+^ T cells were isolated from C57BL/6 mice using magnetic-activated cell sorting (Miltenyi, Bergisch Gladbach, Germany). The cells were cultured in T-cell medium (RPMI 1640 with GlutaMAX, 10% fetal bovine serum, and antibiotics) in 48-well plates precoated with 2 ng/mL anti-CD3 antibody and 1 μg/mL anti-CD28 (BioLegend, San Diego, CA). For human Th17 cell differentiation, 10 ng/mL TGF-β (PeproTech), 50 ng/mL recombinant human IL-6 (PeproTech), 10 ng/mL IL-1β (PeproTech), and 10 ng/mL IL-23 (PeproTech) supplemented with 10 μg/mL anti-human IFN-γ and IL-4 (BioLegend) were added to the T-cell medium. For murine Th17 cell differentiation, cells were cultured for 5 days with 2 ng/mL TGF-β (PeproTech), 50 ng/mL recombinant murine IL-6 (PeproTech), 20 ng/mL IL-1β (PeproTech), and 10 ng/mL IL-23 (BioLegend), plus 10 μg/mL anti-mouse IFN-γ and IL-4 (BioLegend). Naïve CD4^+^ T cells were stimulated under T-cell polarizing condition for 48 h. Afterward, they were co-cultured with MSC added at a 50:1 ratio in a transwell culture system under T-cell polarizing conditions for three days. For metabolite screening, 10 μM metabolites were added 48 h after the initial T-cell polarization. Flow cytometry analysis or Q-PCR was performed three days later.

### Flow cytometry

MSC were harvested 24 h after the addition of mouse lymph node homogenate to the MSC culture medium. Subsequently, MSC labeled with anti-human CD105 and CCR7 antibodies were subjected to subsequent analysis. Lymphocytes were stimulated with cell stimulation cocktail (1:500, Thermo Fisher, Waltham, MA) for 4 h. Dead cells were excluded with the Zombie Aqua™ Fixable Viability Kit (BioLegend). After incubation with Fc receptor blocking antibody (BioLegend), the cell suspension was stained with fluorescence-conjugated antibodies against surface cell markers. After fixation and permeabilization with BD Cytofix/Cytoperm kit (BD Biosciences, San Jose, CA) or transcription factor staining buffer set (BD Biosciences), the cells were intracellularly stained for cytokines or transcription factors. Flow cytometric analysis was performed on CytoFLEX S (Beckman, Beckman, CA) and the data were analyzed by FlowJo (FlowJo, LLC, Ashland, OR).

All antibodies used for flow cytometry were obtained from BioLegend (L/D 423102, anti-human CD105 323203, anti-human CCR7 353213, anti-human CD4 300529, anti-human CD45 304005, anti-human IFN-γ 506506, anti-human IL-17A 512333, anti-mouse CD45 103105, anti-mouse CD4 100406, anti-mouse CD25 102008, anti-mouse IL-4 504103, anti-human IL-17A 560184), BD Biosciences (anti-mouse CD25 561038, anti-mouse IFN-γ 554412, anti-human FOXP3 320013) and eBioscience (anti-RORγ(t) 61-6981-80).

### RNA-seq and analysis

MSC were co-cultured with PBMCs from psoriatic patients at a ratio of 1:50 for 48 h using a transwell culture system. After incubation, MSC were harvested, and total RNA was extracted. RNA sequencing was conducted by Novogene Co., Ltd. (Beijing, China) using the Illumina NovaSeq platform. Differential expression gene (DEGs) analysis was performed with DESeq2, with significance set at an adjusted *P* value ≤ 0.05 and a fold change ≥1.5. Functional analysis, including Gene Ontology (GO) annotation, Kyoto Encyclopedia of Genes and Genomes (KEGG) analysis, and protein–protein interaction analysis was carried out to clarify biological functions.

### Transfection of MSC

MSC were transfected with *IL4I1* overexpression plasmid and siRNA via a recombinant lentivirus. The cloning vector was PGMLV-CMV-MCS-EF1-ZsGreen1-T2A-Puro. The best siRNA sequence was selected through immunoblotting. The *IL4I1* siRNA sequences were 5′-GAAGUGAAGCUGCGCAACUAU-3′ (forward) and 5′-AUAGUUGCGCAGCUUCACUUC-3′ (reverse).

### LC-MS/MS

Standard curves were established with 1-methyltryptophan (Sigma Aldrich) as the internal standard. LAAO downstream metabolites in serum or tissues were extracted using methanol containing the internal standard. Ultra-performance LC system (Shimadzu, Kyoto, Japan) coupled to an AB SCIEX QTRAP^®^ 6500 (Applied Biosystems, Waltham, MA) was used for analysis. Separation was achieved on an XSelect HSS T3 column (3.5 μm, 4.6 mm × 150 mm, Waters, Milford, MA) with a flow rate of 0.5 mL/min at 40 °C. The binary solvent system consisted of 0.1% formic acid in H_2_O (phase A) and methanol (phase B). Chromatograms and mass spectral data were processed by Analyst 1.5.2 (Applied Biosystems).

### Immunofluorescence staining

We used the following primary antibodies for immunofluorescence staining: rabbit anti-CCL21 antibody (Abcam, Cambridge, UK, ab231116; diluted 1:100), high endothelial venule marker monoclonal antibody (MECA-79), Alexa Fluor™ 488 (Thermo Fisher, 53-6036-82; diluted 1:250), rat anti-LYVE1 antibody (Thermo Fisher, 14-0443-82; diluted 1:200), rabbit anti-podoplanin antibody (Abcam, ab256559, diluted 1:200), rabbit anti-CD3 antibody (Abcam, ab231775; diluted 1:200), rabbit anti-CD4 antibody (Abcam, ab183685; diluted 1:200), rabbit anti- RORγt (Abcam, ab207082; diluted 1:200) and mouse anti-Foxp3 antibody (Santa Cruz, Dallas, Tx, sc-53876; diluted 1:50), and rabbit anti-LAAO antibody (Abcam, ab222102; diluted 1:100). To reveal primary antibodies, we used the following secondary antibodies: goat anti-rabbit Alexa Fluor 647, donkey anti-rat Alexa Fluor 488, goat anti-mouse Alexa Fluor 594, and donkey anti-rabbit Alexa Fluor 488 (A-21244, A-21208, A-11005, and A-21206, Thermo Fisher; diluted 1:500).

Lymph node or skin specimens were fixed in 4% paraformaldehyde for 24 h and embedded in Tissue-Tek^®^ O.C.T. Compound (Sakura, Torrance, CA). Tissue sections were prepared using a Leica cryostat CM1950 (Leica Biosystems, Wetzlar, Germany) and blocked with 5% donkey serum. For immunofluorescence staining, primary antibodies were incubated overnight at 4 °C. After washing with PBS, secondary antibodies were incubated for one hour. The slices were subsequently stained with DAPI (Beyotime, Shanghai, China) and mounted in ProLong^®^ Gold Antifade Reagent (Cell Signaling Technology, Danvers, MA). Fluorescence images were acquired using an FV3000 confocal laser scanning microscope (Olympus, Tokyo, Japan). Positive cells were quantified using ImageJ and CellProfiler.

For lymph node transparency, the CUBIC protocol was used [55]. The fixed lymph nodes were immersed in CUBIC-1 reagent and CUBIC-2 reagent respectively to achieve clearing. Mouse CD4 was subsequently stained. Images of the lymph nodes were acquired with a two-photon microscope (Olympus) with a 10X objective Lens and a z-step of 3 mm. Background subtraction was performed with Imaris 9.0 (Bitplane, Zurich, Switzerland).

### LAAO enzyme activity assays

LAAO enzyme activity was measured by H_2_O_2_ generation using Amplex UltraRed [48] (AUR, Thermo Scientific). The cell lysate, supernatant and lymph node homogenate were reacted with a mixture containing 1 U/mL horseradish peroxidase, 50 mM AUR, and 1 mM phenylalanine, tyrosine and tryptophan in 50 mM sodium phosphate, pH 7.0, in a black 96-well microplate. Standard curves were established using gradient dilutions of H_2_O_2_. After incubation at 37 °C in the dark, fluorescence was measured at 530 nm excitation and 590 nm emission wavelengths.

### Dual-luciferase reporter gene assay

We constructed pGL3 luciferase reporter plasmids with either the wild-type or mature *IL4I1* promoter. A DNA mixture containing the PGL3-*IL4I1*-promoter luciferase reporter plasmid and a control pRL-TK Renilla luciferase reporter plasmid was transfected with Lipofectamine 3000 (Thermo Scientific) into HEK293T cells. The *IKKβ* plasmid was co-transfected with the luciferase reporter plasmid, and luciferase activity was measured 4 h later using a dual-luciferase reporter kit (Promega, Madison, WI).

### Immunoblotting

Cells were collected and lysed on ice, followed by centrifugation at 4 °C for 10 min. The supernatant was denatured and loaded on SDS-PAGE for separation and electro-transferred to a PVDF membrane (Bio-Rad). The membranes were blocked and subsequently probed with primary antibodies against LAAO (ab222102, Abcam; diluted 1:3000), DYKDDDDK tag polyclonal antibody (20543-1-AP, Proteintech; diluted 1:3000) and GAPDH (ab181602, Abcam; diluted 1:3000) overnight at 4 °C. The blots were washed and incubated with the appropriate HRP-conjugated secondary antibodies (Bioworld, Nanjing, China). A ChemiDoc XRS system (Bio-Rad) was used to analyze the blots.

### ELISA assays and cytometric bead array

CCL21 concentration in tissue homogenate and cell supernatant were measured using ELISA kits (Elabscience, Wuhan, China). LAAO concentrations in cell lysate and supernatant were measured using ELISA kits (XLPCC, Shanghai, China). Concentration of TNF-α and IL-17A in mouse serum were measured with cytometric bead array (BD Biosciences). We detected the amounts of these cytokines and protein according to the manufacturer’s instructions.

### Statistical analysis

The data are presented as the mean ± standard error of the mean (SEM) and were analyzed using GraphPad Prism 9 (GraphPad Software, La Jolla, CA). The number of biological replicates is indicated in the figure legends. Student’s *t* test with a 95% confidence interval was used to analyze the differences between two groups. Analysis of experiments with more than two groups was performed using one-way analysis of variance (ANOVA) for multiple comparisons.

## Supplementary information

Supplemental Material

Video S1

Video S2

Video S3

Table S2

original full-length Western blots

## Data Availability

All data reported in the paper are included in the manuscript or are available in the Supplemental material.
