# Supplementary material for: Mesenchymal stromal cells restrain the Th17 cell response via L-amino-acid oxidase within lymph nodes
Source: Cell Death Dis. 2024 Sep 2;15(9):640. doi: 10.1038/s41419-024-07024-7 (PMC11383963; doi:10.1038/s41419-024-07024-7)
Supplement: Supplementary file 1 — Supplemental Material [file 41419_2024_7024_MOESM1_ESM.docx]

**Supplemental Material**

**Table S1. Pharmacokinetic parameters of MSC in different tissues of normal mice and IMQ-induced psoriatic mice.**

| Unit | | AUC_last_  (Cells/mg×h) | T_max_  (h) | C_max_  (Cells/mg) |
| --- | --- | --- | --- | --- |
| Lymph  Node | Normal | 2500.8 | 72.0 | 34.5 |
|  | Psoriasis | 8314.9 | 24.0 | 142.1 |
| Skin | Normal | 87.5 | 24.0 | 2.8 |
|  | Psoriasis | 208.1 | 24.0 | 3.5 |
| Lung | Normal | 19186.7 | 1.0 | 1799.8 |
|  | Psoriasis | 23360.2 | 1.0 | 1550.8 |
| Liver | Normal | 4422.1 | 1.0 | 85.6 |
|  | Psoriasis | 2619.9 | 1.0 | 48.4 |
| Spleen | Normal | 516.2 | 1.0 | 12.6 |
|  | Psoriasis | 517.1 | 1.0 | 11.0 |
| Kidney | Normal | 760.5 | 6.0 | 12.0 |
|  | Psoriasis | 342.0 | 6.0 | 9.2 |

**
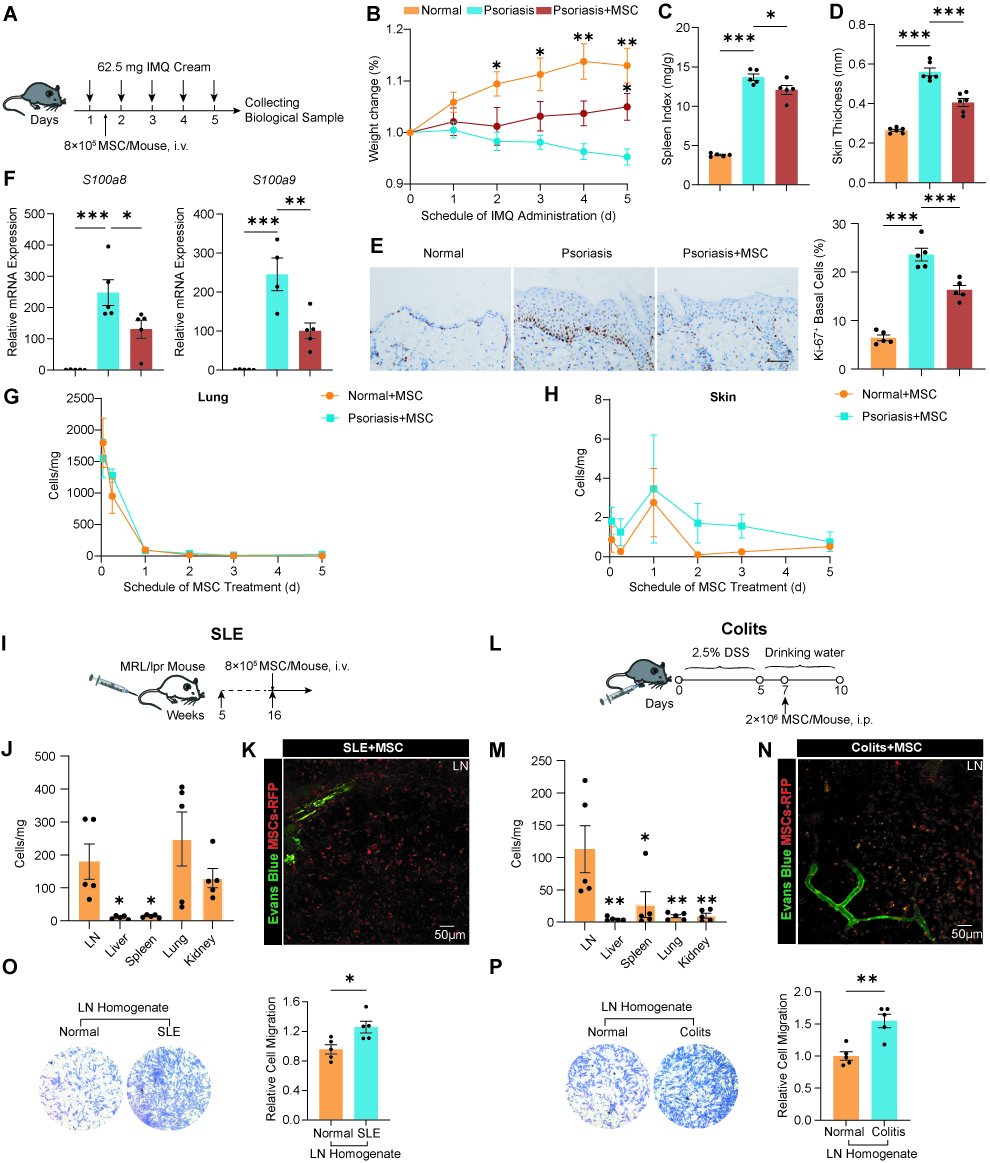
**

**Figure S1.** **MSC exhibit a homing tendency toward lymph nodes in mice with autoimmune diseases. (A) C57BL/6 mice were treated with IMQ for five days, and on the first day, 8×10^5^ human umbilical cord MSC were intravenously injected.** (B) Daily monitoring of weight changes recorded over a 6-day period (n=5). Significant differences compared to psoriatic mice. (C and D) Measurement of the spleen index (C) and skin thickness (D) on day five (n=5). (E) Immunohistochemical staining of Ki-67 in lesional skin with quantification of Ki-67-positive cells in the epidermis (n=5). Scale bar, 100 μm. (F) Keratinocyte differentiation markers in the psoriatic skin of mice were analyzed using Q-PCR (n=5). (G and H) Dynamic profiles of MSC engraftment in the lungs (G) and skins (H) of normal mice and psoriatic mice determined via Q-PCR at the indicated time points (n=5). Significant differences compared to normal mice. (I) Sixteen-week-old MRL/lpr mice received an intravenous infusion of 8×10^5^ MSC through the tail vein. (J) Tissue distribution of MSC in SLE mice was measured by Q-PCR twenty-four hours after MSC administration (n=5). Significant differences in comparison with lymph nodes. (K) Representative images of RFP-MSC captured by intravital multiphoton (IVIM) imaging in the inguinal lymph nodes of SLE mice twenty-four hours after MSC administration. Scale bar, 50 μm. (L) C57BL/6 mice were fed 2.5% dextran sodium sulfate polymers (DSS) in drinking water for five days, followed by normal drinking water. On day seven, 2×10^6^ MSC were intraperitoneally injected. (M) Tissue distribution of MSC in colitis mice was measured with Q-PCR twenty-four hours after MSC administration (n=5). Significant differences compared to lymph nodes. (N) Representative images of RFP-MSC captured by IVIM imaging in the inguinal lymph nodes of mice with colitis twenty-four hours after MSC administration. Scale bar, 50 μm. (O and P) Representative images and quantification of MSC recruited by homogenates of lymph nodes from SLE mice (O) and colitis mice (P), respectively, in a transwell culture system (n=5). The data are presented as the means ± SEMs. **P<0.05, **P<0.01 and ***P<0.001.* MSC: mesenchymal stromal cell, IMQ: imiquimod, LN: lymph node, SLE: systemic lupus erythematosus, DSS: dextran sodium sulfate polymers.


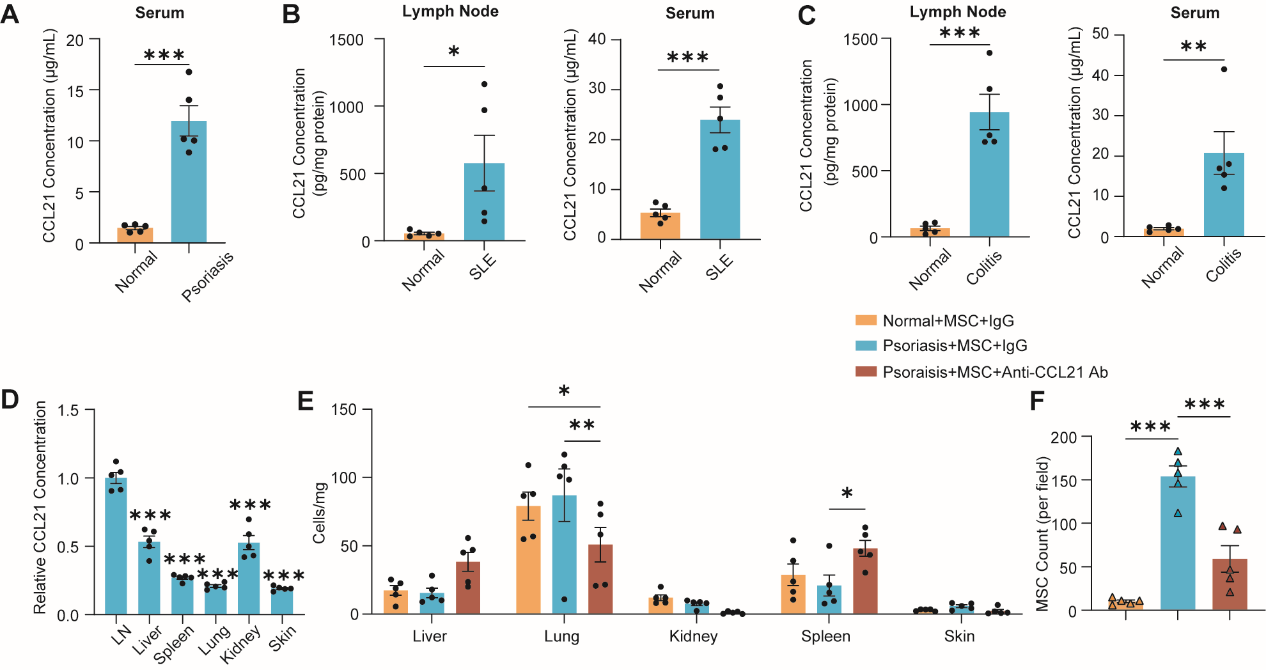


**Figure S2.** **CCL21-CCR7 axis mediates MSC homing to lymph nodes.** (A) Measurement of the CCL21 concentration in the serum of normal mice and IMQ-induced psoriatic mice (n=5). (B and C) Assessment of the CCL21 concentration in the lymph nodes and serum of SLE mice (B) and colitis mice (C) (n=5). (D) Relative CCL21 concentration in different tissues of psoriatic mice (n=5). Significant differences in comparison with lymph nodes. (E) Quantification of MSC in different tissues of psoriatic mice pretreated with an anti-CCL21 antibody twenty-four hours after MSC administration by Q-PCR (n=5). (F) Quantification of MSC-RFP in inguinal lymph nodes from psoriatic mice pretreated with anti-CCL21 antibody by IVIM imaging (n=5). The data are presented as the means ± SEMs. **P<0.05, **P<0.01 and ***P<0.001.*

**
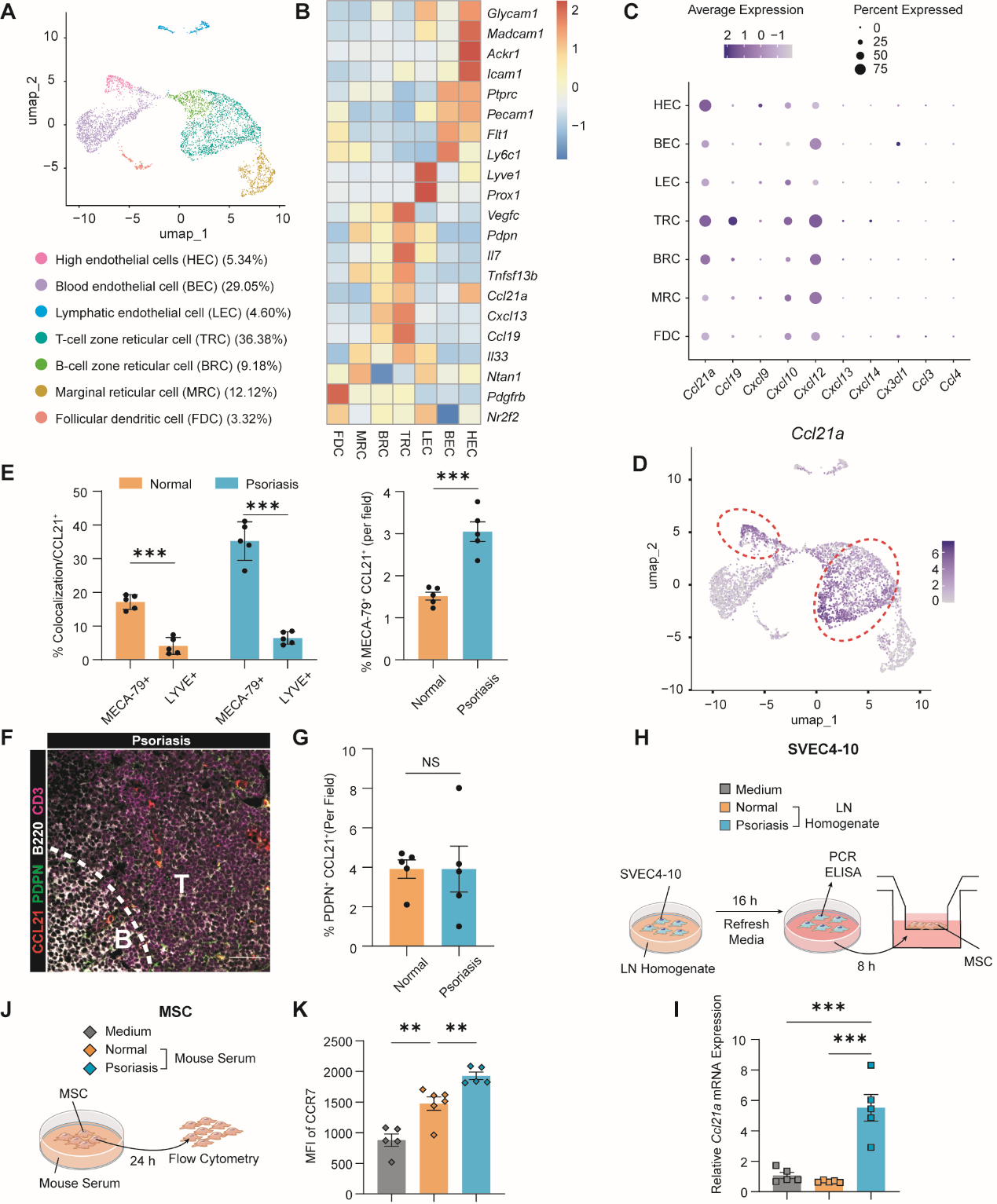
**

**Figure S3. The expression of CCL21-CCR7 axis was upregulated in psoriatic mice.** (A-D) Single-cell RNA-seq analysis of FACS-isolated CD45^-^CD24^-^ skin-draining peripheral lymph node cells from C57BL/6 mice. All the data were obtained from the GEO database (GSM5819066). (A) UMAP plot displaying all cell clusters. (B) Heatmap of the expression of segregating genes. (C) Air bubble diagram illustrating the mRNA expression of chemokines in cells as indicated. (D) UMAP plot displaying the expression of *Ccl21a* in lymph nodes. (E) Quantification of the CCL21 positive region and its colocalization with MECA-79 and LYVE conducted using CellProfiler. (n=5). (F) Immunofluorescence staining of peripheral lymph nodes for CCL21, stromal cells (PDPN), B cells (B220) and T cells (CD3). Scale bar, 50 μm. (G) Quantification of the CCL21-positive region and its colocalization with PDPN in the peripheral lymph nodes from normal mice and psoriatic mice (n=5). (H) Illustration of SVEC4-10 cells being stimulated with homogenates of the lymph nodes from normal mice and psoriatic mice. After sixteen hours, the homogenate was removed, and the culture media was refreshed. (I) Relative gene expression of *Ccl21a* in SVEC-4-10 cells after stimulated with homogenates of lymph nodes. (J) Illustration of MSC being stimulated with serum from normal mice and psoriatic mice. (K) CCR7 expression in MSC determined by flow cytometry (n=5). The data are presented as the means ± SEMs. **P<0.05, **P<0.01 and ***P<0.001.* HEC: high endothelial cell, TRC: T-cell zone reticular cell, BRC: B-cell zone reticular cell, FRC: fibroblastic reticular stromal cell, BEC: blood endothelial cell, LEC: lymphatic endothelial cell, FDC: follicular dendritic cell, MRC: marginal reticular cell.


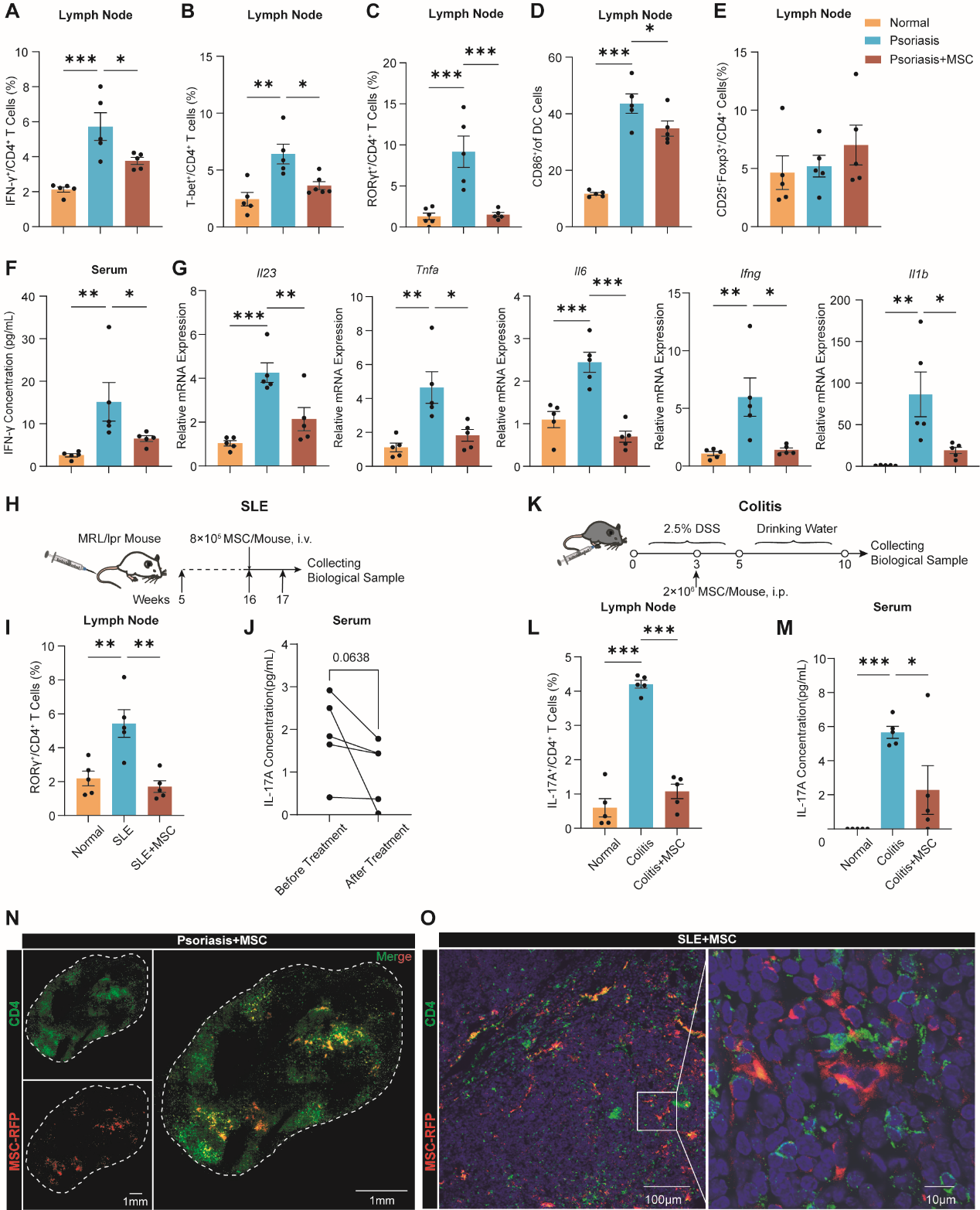


**Figure S4. MSC treatment restrains the Th17 cell response and dampens inflammation.** (A-E) Populations of Th1 cells (A and B), Th17 cells (C), mature DCs (D), and Treg cells (E) in the peripheral lymph nodes of psoriatic mice quantified using flow cytometry five days after MSC treatment (n=5). (F) Serum IFN-γ levels in psoriatic mice (n=5). (G) Inflammatory factors in the psoriatic skin of mouse (n=5). (H) The scheme illustrates the MSC treatment for SLE mice. (I) The population of Th17 cells in the peripheral lymph nodes of SLE mice seven days after MSC treatment (n=5). (J) Serum levels of IL-17A in SLE mice before and seven days after MSC treatment (n=5). (K) The scheme illustrates the MSC treatment for colitis mice. (L) The population of Th17 cells in the peripheral lymph nodes of colitis mice seven days after MSC treatment (n=5). (M) Serum levels of IL-17A in mice with colitis seven days after MSC treatment (n=4). (N) Z-stack projection of images. Psoriatic mice received intravenous injections of MSC labeled with RFP. After twenty-four hours, the mice were sacrificed, and the lymph nodes were transparentized and stained for CD4^+^ T cells. Scale bar, 1 mm. (O) Distribution of MSC-RFP and immunofluorescence of CD4 in peripheral lymph nodes from SLE mice twenty-four hours after administration. The white box indicates the region of interest. Scale bars, 100 μm and 10 μm. The data are presented as the means ± SEMs. **P<0.05, **P<0.01 and ***P<0.001.*


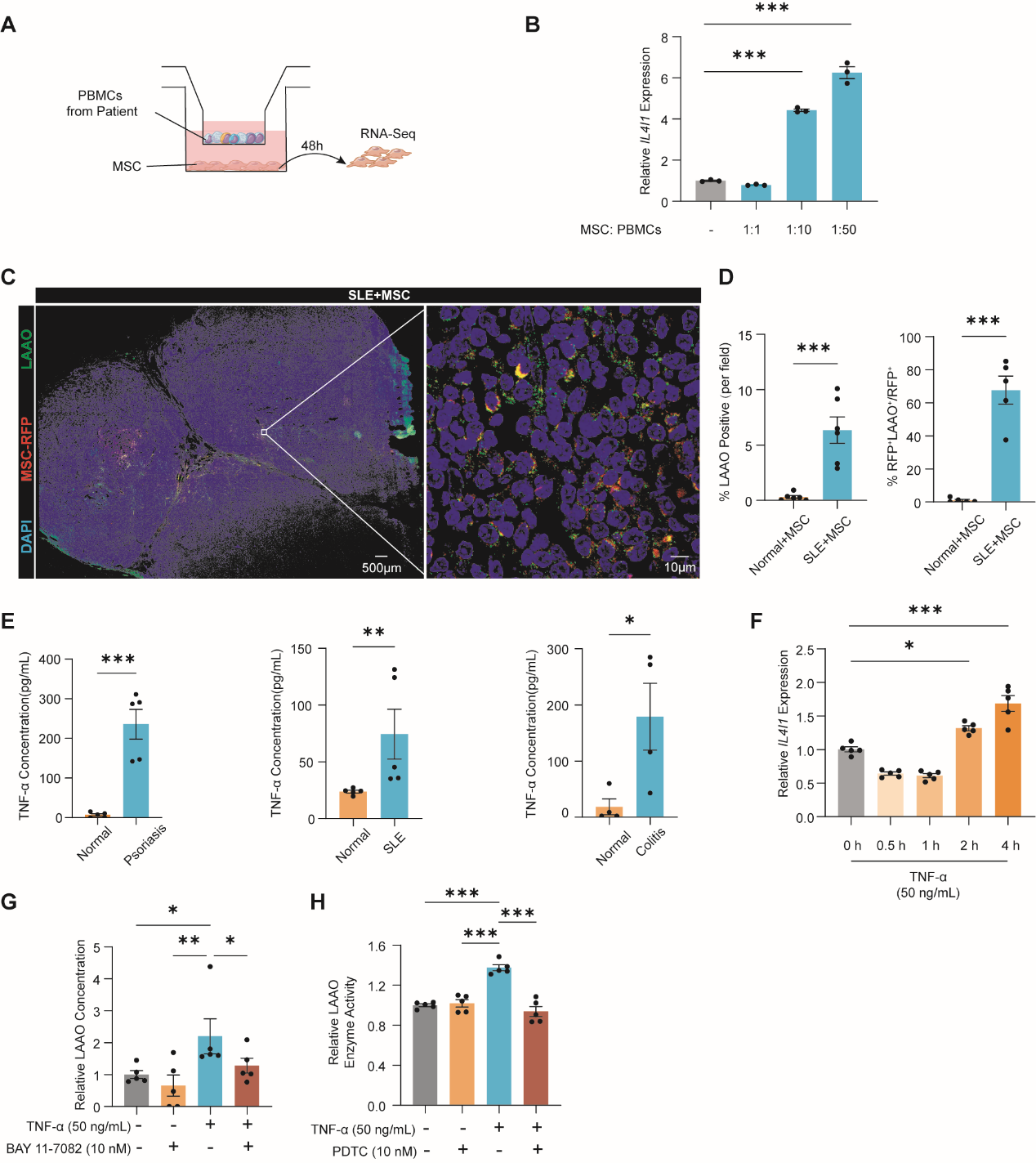


**Figure S5. MSC secrete LAAO in response to inflammation through the NF-κB pathway.** (A) Illustration of the scheme depicting MSC co-cultured with PBMCs derived from psoriatic patients at a ratio of 1:50 for forty-eight hours, employing a transwell culture system. Following this incubation period, the MSC were harvested for RNA sequencing. (B) Assessment of *IL4I1* expression in MSC co-cultured with PBMCs at various ratios (n=3). (C) Immunofluorescence colocalization analysis of human LAAO with MSC-RFP in the lymph nodes of SLE mice twenty-four hours after MSC infusion. Scale bars, 500 μm and 10 μm. (D) Quantification of LAAO immunofluorescence and colocalization of LAAO with MSC-RFP using CellProfiler in the lymph nodes of SLE mice (n=5). (E) Serum concentrations of TNF-α in psoriatic mice, SLE mice and colitis mice (n=4-5). (F) *IL4I1* gene expression in MSC following TNF-α treatment over time (n=5). (G) Protein levels of LAAO in the supernatant of MSC after treatment with TNF-α for twenty-four hours, in addition to BAY 11-7082 (n=5). (H) LAAO enzyme activity in the supernatant of MSC after treatment with TNF-α for twenty-four hours, in addition to the NF-κB inhibitor PDTC (n=5). The data are presented as the means ± SEMs. **P<0.05, **P<0.01 and ***P<0.001.* PBMC: peripheral blood mononuclear cell.


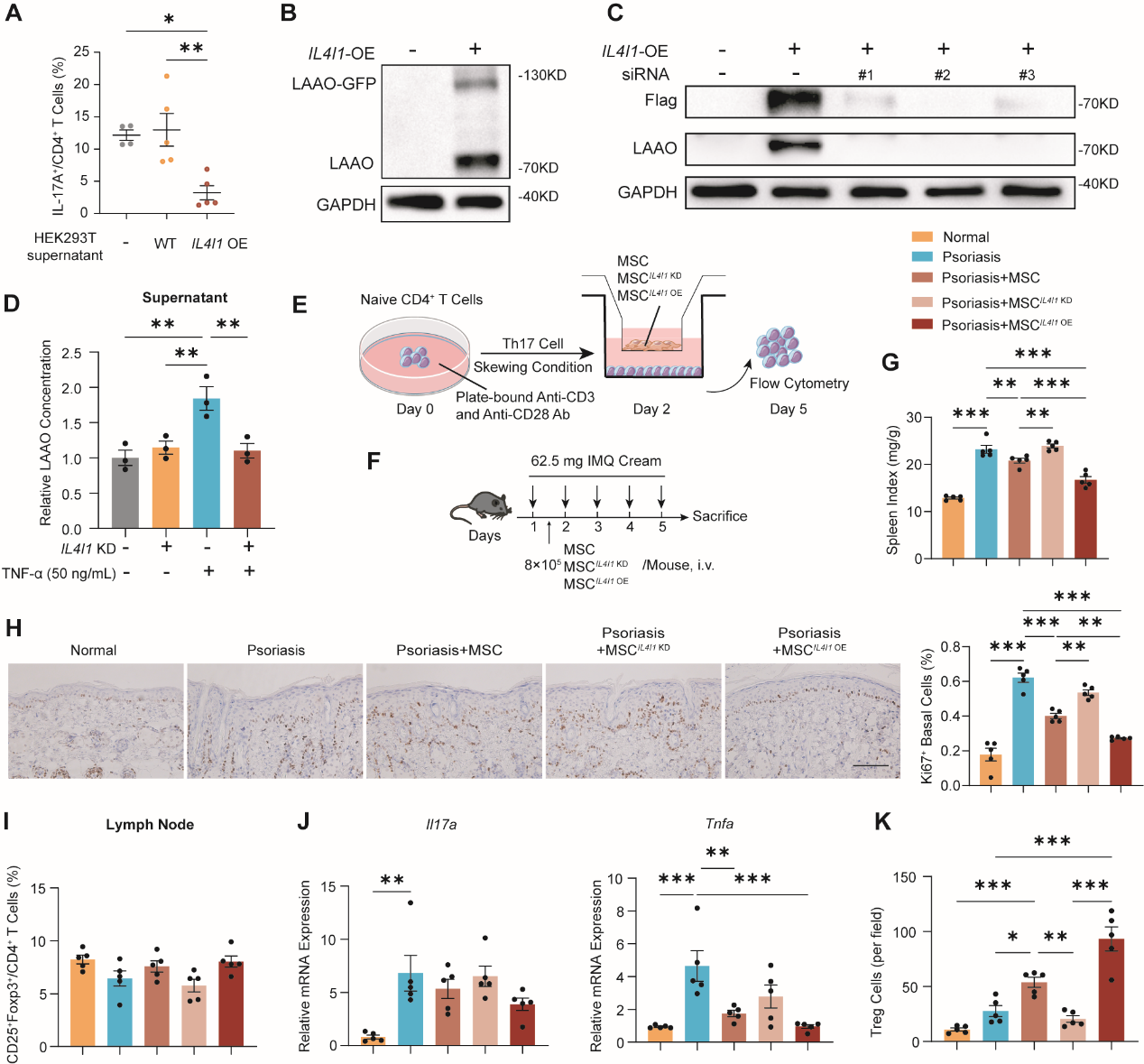


**Figure S6.** **LAAO-dependent suppression of Th17 cells by MSC.** (A) Flow cytometry analysis of cytokine staining in murine CD4^+^ T cells differentiated under Th17 cell polarizing condition containing the supernatants of HEK293T cells with ectopic human LAAO (n=4-5). (B) Immunoblotting of LAAO in MSC overexpressing *IL4I1* via a lentiviral vector. (C) Immunoblotting analysis of LAAO protein levels in HEK293T cells transfected with different small interfering RNAs (siRNAs) targeting *IL4I1* (#1, #2, #3). (D) Relative concentration of LAAO in MSC with *IL4I1* knockdown (MSC*^IL4I1^* ^KD^) via a lentiviral vector based on the #2 siRNA (n=3). (E) Experimental schematic of murine T cells co-cultured with MSC in which *IL4I1* was knocked down (MSC*^IL4I1^* ^KD^) or overexpressed (MSC*^IL4I1^* ^OE^). (F) Experimental schematic of psoriatic mice intravenously injected with 8×10^5^ MSC featuring *IL4I1* knockdown or overexpression. (G) Spleen index of psoriatic mice on day five. (H) Immunohistochemical staining of Ki-67 in lesional skin and quantification of Ki-67 positive cells in the epidermis (n=5). Scale bar, 100 μm. (I) Percentage of Treg cells in the peripheral lymph nodes of psoriatic mice treated with MSC featuring *IL4I1* knockdown or overexpression (n=5). (J) Inflammatory factors in the psoriatic skin of mice(n=5). (K) Quantification of infiltrated Treg cells in lesional skin tissue sections. The data are presented as the means ± SEMs. **P<0.05, **P<0.01 and ***P<0.001.*

Video S1. Video of MSC within the blood vessels of the inguinal lymph nodes from psoriatic mice, captured by IVIM imaging 2 hours after MSC treatment.

Video S2. 3D reconstruction of MSC within the blood vessels of inguinal lymph nodes from psoriatic mice, captured by IVIM imaging 6 hours after MSC treatment.

Video S3. 3D reconstruction of the localized magnification of MSC in the inguinal lymph nodes from psoriatic mice, captured by IVIM imaging twenty-four hours after MSC treatment.
