# Supplementary material for: Mesenchymal stromal cells restrain the Th17 cell response via L-amino-acid oxidase within lymph nodes
Source: Cell Death Dis. 2024 Sep 2;15(9):640. doi: 10.1038/s41419-024-07024-7 (PMC11383963; doi:10.1038/s41419-024-07024-7)
Supplement: Supplementary file 6 — original full-length Western blots [file 41419_2024_7024_MOESM6_ESM.docx]

**Figure S5B**

GAPDH


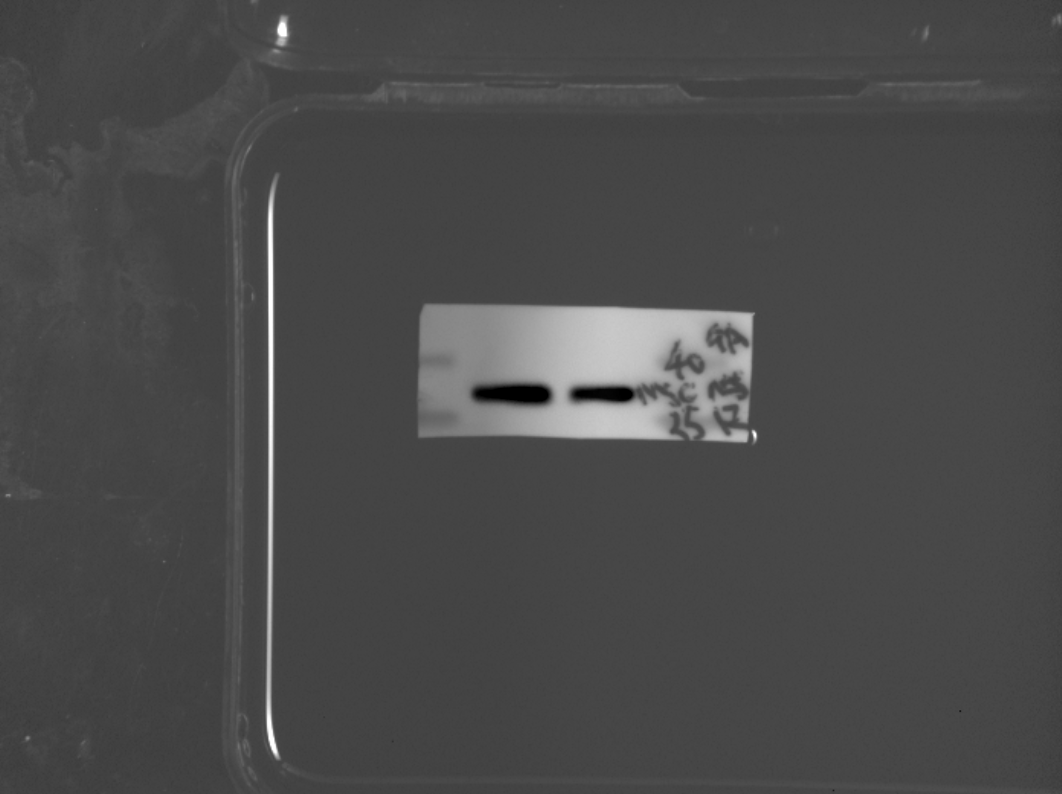


Control MSCs*^IL4I1^* ^OE^


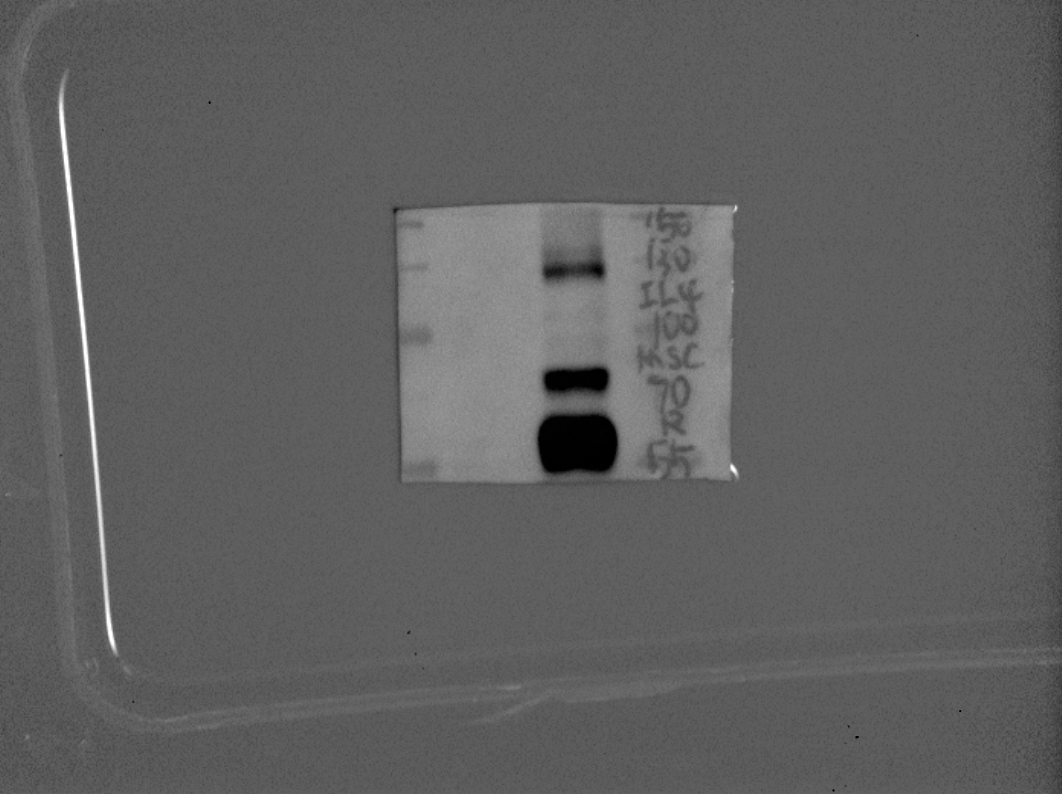
LAAO

Control MSCs*^IL4I1^* ^OE^

**Figure S5C**

GAPDH


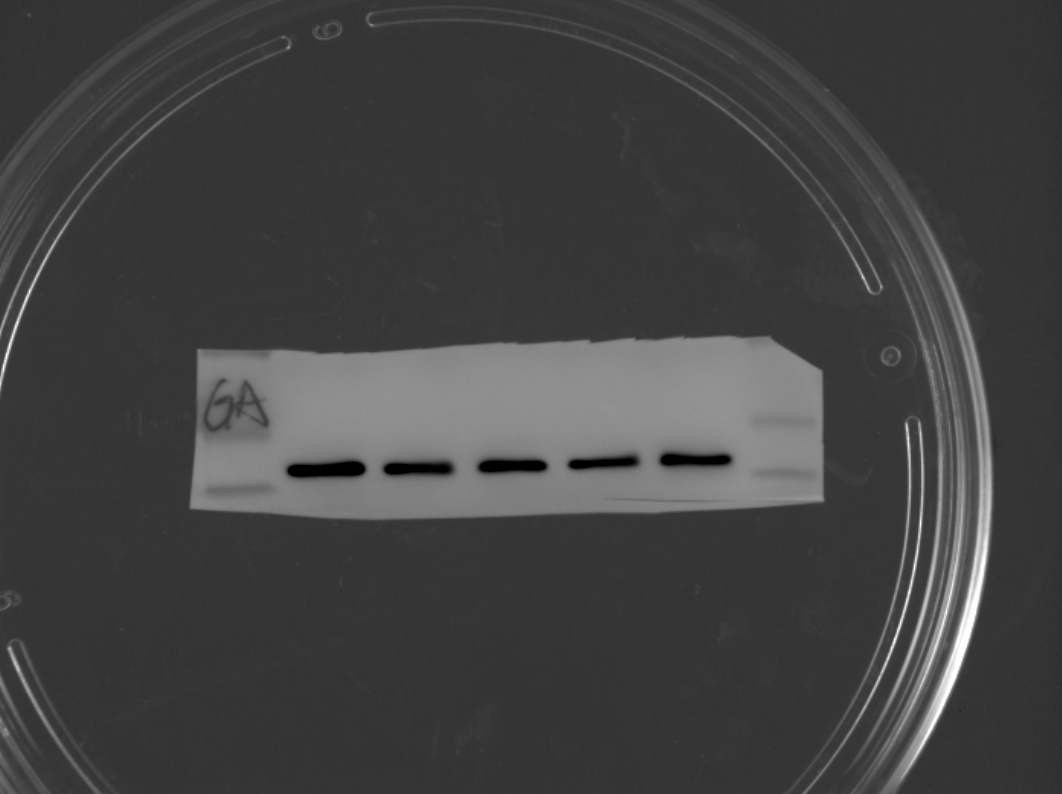


40

OE - + + + +

SiRNA - - #1 #2 #3

LAAO


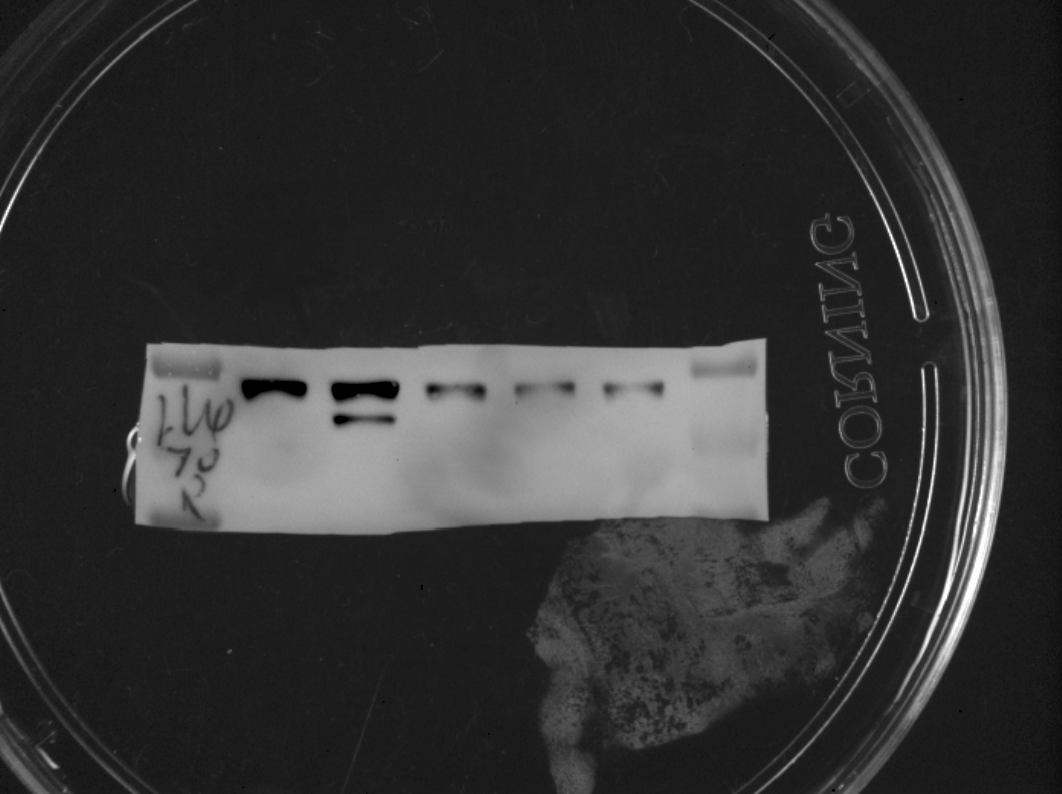


OE - + + + +

SiRNA - - #1 #2 #3

70

Flag


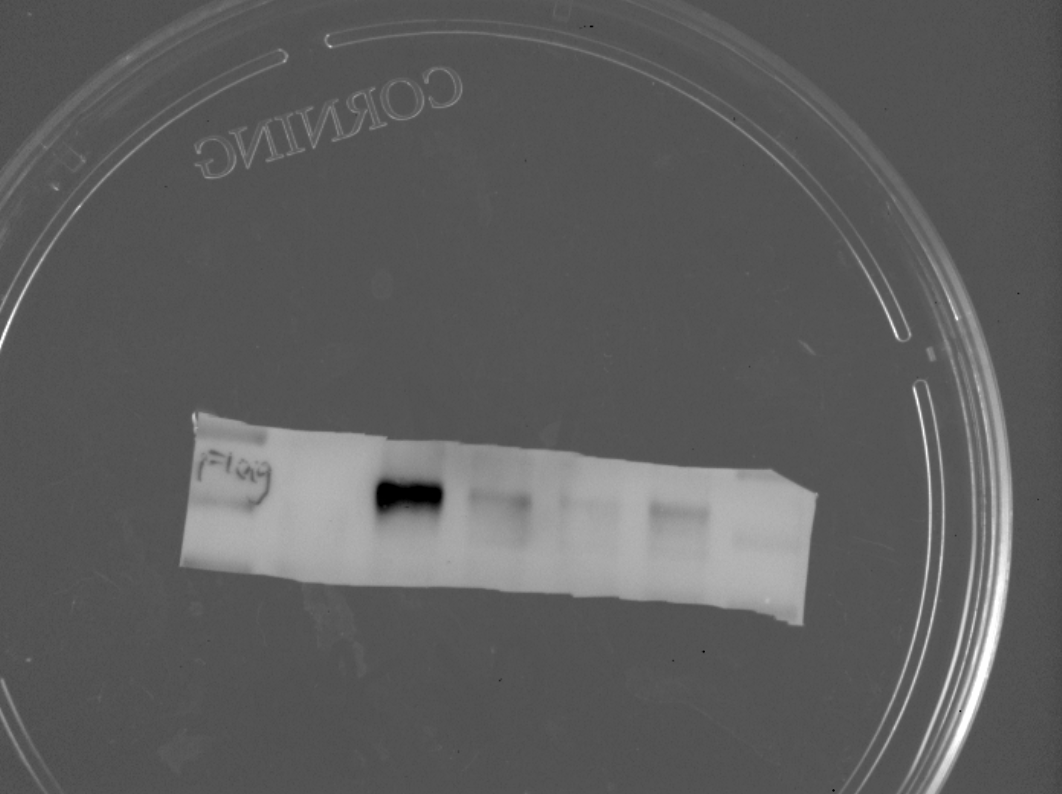


OE - + + + +

SiRNA - - #1 #2 #3

70
